# Extracellular wax ester biosynthesis by staphylococcal lipases detoxifies skin fatty acids and shapes interspecies competition

**DOI:** 10.1093/ismejo/wrag148

**Published:** 2026-06-11

**Authors:** Justine Camus, Christian D Freeman, Xuanheng Hu, Aaron Komnik, Rebekah L Casey, David T Brewer, Soobin Yang, Kelly M Hines, Arnaud Kengmo Tchoupa

**Affiliations:** Interfaculty Institute of Microbiology and Infection Medicine Tübingen, Chair of Infection Biology, University of Tübingen, 72076 Tübingen, Germany; Cluster of Excellence EXC 2124 Controlling Microbes to Fight Infections, University of Tübingen, 72076 Tübingen, Germany; German Centre for Infection Research (DZIF), Partner Site Tübingen, 72076 Tübingen, Germany; Department of Chemistry, University of Georgia, Athens, Georgia 30602, United States; Interfaculty Institute of Microbiology and Infection Medicine Tübingen, Chair of Infection Biology, University of Tübingen, 72076 Tübingen, Germany; Cluster of Excellence EXC 2124 Controlling Microbes to Fight Infections, University of Tübingen, 72076 Tübingen, Germany; German Centre for Infection Research (DZIF), Partner Site Tübingen, 72076 Tübingen, Germany; Interfaculty Institute of Microbiology and Infection Medicine Tübingen, Chair of Infection Biology, University of Tübingen, 72076 Tübingen, Germany; Cluster of Excellence EXC 2124 Controlling Microbes to Fight Infections, University of Tübingen, 72076 Tübingen, Germany; German Centre for Infection Research (DZIF), Partner Site Tübingen, 72076 Tübingen, Germany; Department of Chemistry, University of Georgia, Athens, Georgia 30602, United States; Department of Chemistry, University of Georgia, Athens, Georgia 30602, United States; Interfaculty Institute of Microbiology and Infection Medicine Tübingen, Chair of Infection Biology, University of Tübingen, 72076 Tübingen, Germany; Cluster of Excellence EXC 2124 Controlling Microbes to Fight Infections, University of Tübingen, 72076 Tübingen, Germany; German Centre for Infection Research (DZIF), Partner Site Tübingen, 72076 Tübingen, Germany; Department of Chemistry, University of Georgia, Athens, Georgia 30602, United States; Interfaculty Institute of Microbiology and Infection Medicine Tübingen, Chair of Infection Biology, University of Tübingen, 72076 Tübingen, Germany; Cluster of Excellence EXC 2124 Controlling Microbes to Fight Infections, University of Tübingen, 72076 Tübingen, Germany; German Centre for Infection Research (DZIF), Partner Site Tübingen, 72076 Tübingen, Germany

**Keywords:** antimicrobial fatty acids, lipase, glycerol ester hydrolase, wax ester, *Staphylococcus aureus*, esterification, biofilm, interspecies competition, collective interspecies resistance, coagulase negative staphylococci

## Abstract

To maintain its barrier function, human skin requires lipids, including cholesterol, ceramides, free fatty acids, and wax esters. In contrast to other skin lipids, wax esters remain largely unexplored as metabolites of the skin microbiome. Recently, we discovered that *Staphylococcus aureus* utilises its lipase Lip2 to detoxify antimicrobial fatty acids (AFAs) by esterification with cholesterol. The observed promiscuity of Lip2 and the presence of similar lipases among skin staphylococci prompted the search for novel substrates and products. Here, we identify *S. aureus* Lip2 and Lip1, *Staphylococcus simulans* SsL, and *Staphylococcus epidermidis* GehD and GehC as lipases capable of metabolizing wax esters. These lipases degraded wax esters into AFAs and fatty alcohols, and, except for GehC, shared the ability to esterify AFAs with fatty alcohols, thereby generating wax esters. In monocultures of bacteria heterologously expressing staphylococcal lipases, synthesis of wax esters mirrored protection from AFAs by fatty alcohols in planktonic or biofilm settings. In pairwise cocultures, lipases secreted by lipase-proficient *S. aureus*, *S. simulans*, and *S. epidermidis* functioned as public goods, rescuing lipase-deficient mutants. In the absence of detoxifying substrates/lipases, *S. simulans* or *S. epidermidis* outcompeted *S. aureus* when exposed to AFAs. A skin-mimicking high-salt environment increased the resistance of *S. aureus*, but not *S. simulans*, to AFAs, enabling *S. aureus* to match or outcompete *S. simulans* in the presence of AFAs or AFAs plus fatty alcohols, respectively. Collectively, our findings suggest that commensal modulation of skin lipids determines whether niche-specific communities are resilient or permissive to pathogenic invasion by *S. aureus*.

## Graphical Abstract

**Graphical Abstract:**
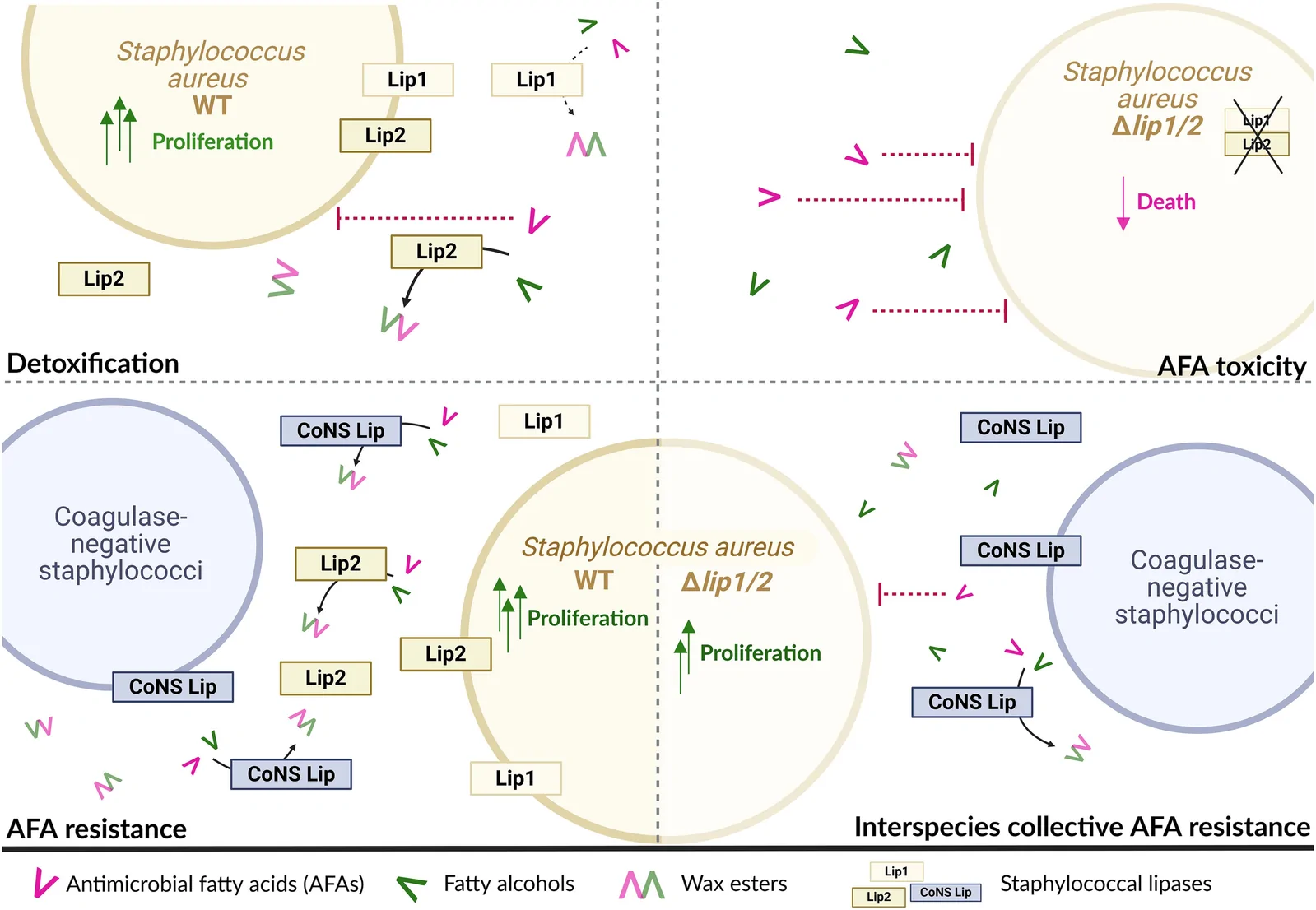

## Introduction

The skin is a multilayered barrier against thermal, mechanical, chemical, and microbial insults [1, 2]. Besides blocking the penetration of undesirable, foreign substances, the skin prevents the leakage of water and solutes [3]. Despite the scarcity of nutrients, the skin is diversely populated by a niche-specific microbiome with key roles in tissue homeostasis [1, 2, 4, 5]. The postulated functions of resident skin microbes include protection against pathogens and modulation of immune responses [6, 7]. The skin microbiome is dominated by bacterial species from three major phyla: Actinobacteria (*Cutibacterium* and *Corynebacterium*), Firmicutes (*Staphylococcus* and *Micrococcus*), and Proteobacteria (Acinetobacter) [5]. In addition to bacteria, the human skin microbiome encompasses fungi and viruses [1].

An aberrant composition of the skin microbiome coincides with various skin disorders. For instance, acne vulgaris is associated with overgrowth of *Cutibacterium acnes* [8]. Contrastingly, *C. acnes* is depleted on the skin of psoriasis patients [9]. Psoriasis lesions are also associated with altered abundance of *Corynebacterium* species [9]. These species change in their relative abundance during ichthyosis [10]. In the case of atopic dermatitis (AD), the skin microbiome is dominated by *S. aureus* [11]. Intriguingly, antimicrobial-producing coagulase-negative staphylococci (CoNS) are rare on AD skin and pervasive on healthy skin [12]. In the hair follicle, *C. acnes* produces cutimycin, which inhibits CoNS and *S. aureus* [13]. The colonisation of human skin by *S. aureus* has also been shown to be hampered by the commensal fungus *Malassezia sympodialis*, presumably via a hydroxy fatty acid [14]. Free fatty acids released from skin surface triacylglycerols by *Corynebacterium accolens* limit human colonisation by *Streptococcus pneumoniae* [15].

Beyond contributing to colonisation resistance, skin lipid remodeling by the microbiome also impacts barrier integrity [16]. For instance, *S. epidermidis*, a ubiquitous commensal of the human skin, utilises its sphingomyelinase to produce ceramides contributing to skin integrity [17]. Glycerol ester hydrolases (Geh) are additional enzymes (lipases) that *S. epidermidis* (GehC and GehD) [18] and *C. acnes* (GehA) [19] utilise to hydrolyze triacyglycerols and release free fatty acids, which contribute to the acid mantle of the skin [5]. Moreover, *C. acnes*’ metabolism generates short-chain fatty acids, potently activating skin lipid synthesis [20]. Another skin commensal, *Roseomonas mucosa*, sheds lipids to induce epithelial repair [21].

Owing to their roles in shaping host–microbe and microbe–microbe interactions, lipids have been extensively studied to decipher skin disorders [22–29]. However, wax esters, sebaceous lipids largely recalcitrant to microbial hydrolysis [30], remain largely overlooked. Our recent discovery [31] that the *S. aureus* lipase Lip2 detoxifies antimicrobial fatty acids (AFAs) via cholesterol esterification raised the possibility that skin bacteria could metabolize wax esters similarly. Here, we utilise physiologically relevant lipid concentrations in various growth media and skin-mimicking conditions to investigate whether and how staphylococcal lipases synthesize and/or hydrolyse wax esters. Our results support a model in which AFAs are more toxic to the opportunistic and metabolically versatile pathogen *S. aureus* compared to CoNS. Consequently, AFAs would enable CoNS to outcompete *S. aureus* in AFA-rich environments such as the skin. AFA detoxification via wax ester production promotes *S. aureus* replication regardless of whether the lipases are from *S. aureus* or CoNS. Thus, we propose that the impact of AFAs on skin microbiomes is governed by microbial lipases and the availability of their detoxifying substrates.

## Materials and methods

### Bacterial strains and assays

All bacterial strains used in this study are listed in Table S1. The Supplementary Information provides detailed protocols for growth, spot, lipase activity, biofilm, and co-cultivation assays, qPCR, as well as the purification of recombinant staphylococcal lipases, lipid extraction, and subsequent analysis via high-performance thin layer chromatography (HPTLC).

### Complementation of lipase-deficient *S. aureus* with lipases of CoNS

To evaluate the activity of lipases from coagulase-negative staphylococci (CoNS), including *S. epidermidis* and *S. simulans*, plasmids with individual lipase genes were constructed. Primers used for cloning are listed in Table S2. Lipase-encoding genes from *S. simulans* ATCC27848 and *S. epidermidis* 1457 were amplified by PCR from extracted genomic DNA (NucleoSpin Microbial DNA kit, Macherey-Nagel). Purified PCR products were inserted into the multicopy pALC2073 vector with a leaky tetracycline-inducible *P_xyl/tetO_* promoter [32] using Thermo Scientific FastDigest restriction enzymes (KpnI and SacI) and the resulting plasmids were transformed into chemically competent *Escherichia coli* IM08B [33]. Successful cloning was confirmed by Sanger sequencing prior to transformation of *S. aureus* USA300 JE2 Δ*lip1/2* by electroporation [34].

### Sample preparation for lipid identification using HILIC-IM-MS

To evaluate whether lipases catalyse the esterification of AFAs with fatty alcohols (FOH) to produce wax esters, recombinant lipases or bacteria were incubated with palmitoleic acid (PA) and palmitoleic alcohol (POH) in 1.5 mL glass vials (Macherey-Nagel GmbH). On one hand, recombinant lipases (1 μg/mL) of *S. aureus* (Lip1 and Lip2), *S. simulans* (SsL) or *S. epidermidis* (GehC and GehD) were incubated with 100 μM PA and 100 μM POH in 0.1 M sodium phosphate buffer (pH 6.0) for 10, 30, 60, 120 min or 24 h. A negative control consisting of buffer supplemented with PA and POH, but lacking lipase was included. On the other hand, bacteria were grown in basic medium supplemented with 300 μM PA and 300 μM POH. After 24 h, cultures were serially diluted in PBS and plated on TSA for colony-forming unit (CFU) enumeration. Culture supernatants and bacterial pellets were separated via centrifugation (4700 *g*, 15 min). Bacterial supernatants were transferred to clean glass vials (Roland Vetter Laborbedarf OHG) while pellets were washed twice, resuspended in water, and transferred to fresh glass vials. Lipid extraction was performed using a modified Bligh and Dyer protocol [35, 36]. Chilled chloroform:methanol (1:2, v/v) was added to samples prior to vortexing for 1 min (samples with recombinant proteins or supernatants) or 5 min (pellets). Phase separation was induced by further addition of water and chloroform prior to centrifugation (2000 × *g* for 10 min at 4°C). The organic phase was collected, transferred to clean 1.5 mL glass vials, and dried using a vacuum concentrator (miVac Quattro Concentrator, Genevac). Dried lipid extracts were stored frozen until further analysis.

### Lipid identification using HILIC-IM-MS

Dried lipid extracts were reconstituted in 0.5 mL of 1:1 chloroform/methanol. Aliquots of the lipid extracts were dried in a vacuum concentrator and diluted in 95% acetonitrile/5% water with 10 mM ammonium acetate (HILIC A Mobile Phase) prior to HILIC-IM-MS analysis. A 10-fold dilution was used for both ionisation modes for the analysis of bacterial and supernatant lipid extracts from 24 h incubations with 300 μM PA/POH and lipid extracts from 10–120 min incubations of recombinant proteins with 100 μM PA/POH. The 24 h incubations of recombinant proteins with 100 μM PA/POH were prepared as 35-fold and 5-fold dilutions for negative and positive modes, respectively. A pooled quality control sample was prepared by combining 5 μL of each sample together into a single vial.

Lipid analyses were performed using a Waters Acquity FTN I-Class Plus ultra-performance liquid chromatography system coupled to the ESI source of a Waters Synapt XS ion mobility-mass spectrometer. A Waters CORTECS HILIC (2.1x100 mm, 1.7 μm) column was used for lipid separations [35, 37]. The mobile phases consisted of 95:5 acetonitrile/water with 10 mM ammonium acetate (HILIC A) and 1:1 acetonitrile/water with 10 mM ammonium acetate (HILIC B). A flow rate of 0.5 mL/min was applied over a 7-min gradient with the following conditions: 0–0.5 min (100% MPB), 0.5–5 min (100–60% MPB), 5–5.5 min (60% MPB), 5.5–6 min (60–100% MPB), 6–7 min (100% MPB). Data were collected over the range of 50–1200 *m/z* in both positive and negative ionisation modes with traveling wave ion mobility separation (wave velocity, 550 m/s; wave height, 40 V; N2 gas flow; 90 mL/min) and post-mobility data-independent tandem MS acquisition (ramped collision energy; 45–60 V for positive and 35–50 V for negative ionisation modes). Lockspray mass correction was performed using leucine enkephalin. Data alignment, feature finding, and normalisation was performed in Progenesis QI (v3.0, Nonlinear Dynamics/Waters). Samples were aligned to the pooled quality control reference and normalisation was performed using the “all compounds” method. Lipids were identified using an accurate mass tolerance of ≤5 ppm against a customised version of LipidPioneer [38], as well as retention time and MS/MS spectral matching against standards. Fatty acids (FA) and phosphatidylglycerols (PG) were reported from negative ESI data as [M-H]^−^ adducts. Wax esters (WE) were reported from positive ESI data as [M + H]^+^ adducts, in agreement with a palmitoleyl palmitoleate WE standard.

### Construction of a marker-less deletion mutant in *S. simulans*

In-frame deletion of the gene encoding the *Staphylococcus simulans* lipase (*ssL*) was created by pIMAY-enabled allelic exchange. The flanking regions (~500 bp) of *ssL* were amplified separately by PCR. The obtained amplicons (upstream and downstream of *ssL*) were fused by overlap extension PCR and cloned into pIMAY [34] using KpnI and SacI restriction sites. This recombinant plasmid was transformed into chemically competent *E. coli* IM08B. Upon purification, control digestion and sequencing, the plasmid was used to transform *S. aureus* PS187 ∆*sauUSI*∆*hsdR* [39] by electroporation. Next, the bacteriophage Φ187 enabled plasmid transfer from this *S. aureus* PS187 mutant to *S. simulans*, as described previously with other CoNS [40]. The *ssL*-deletion pIMAY construct was then integrated into the chromosome of *S. simulans* via homologous recombination upon incubation at a non-permissive temperature for pIMAY replication (37°C). The excision and loss of the plasmid was stimulated by several passages at 30°C (permissive temperature for pIMAY replication) without antibiotics. These cultures were plated onto TSA containing 1 μg/mL anhydrotetracycline (ATC) and incubated for 2 days at 30°C. Large colonies, which were unlikely to be inhibited by ATC-induced expression the *secY* anti-sense RNA on pIMAY, were patched on TSA, TSA + chloramphenicol, and Baird Parker agar (BPA) plates supplemented with egg yolk tellurite prior to overnight growth at 37°C. Chloramphenicol-susceptible clones, which displayed no lipase activity on BPA plates were grown in TSB overnight at 37°C in order to confirm *ssL* deletion upon chromosomal DNA purification, PCR, and sequencing.

### Statistical analysis

All statistical analyses were performed using GraphPad Prism version 10.6.1. The specific statistical tests used are indicated in the corresponding figure legends. A *P* value <0.05 was considered statistically significant. Ordinary one-way analysis of variance (ANOVA) or two-way ANOVA was used wherever appropriate.

## Results

### Lipase-dependent protection of *S. aureus* from AFA toxicity by fatty alcohols

**Figure 1:**
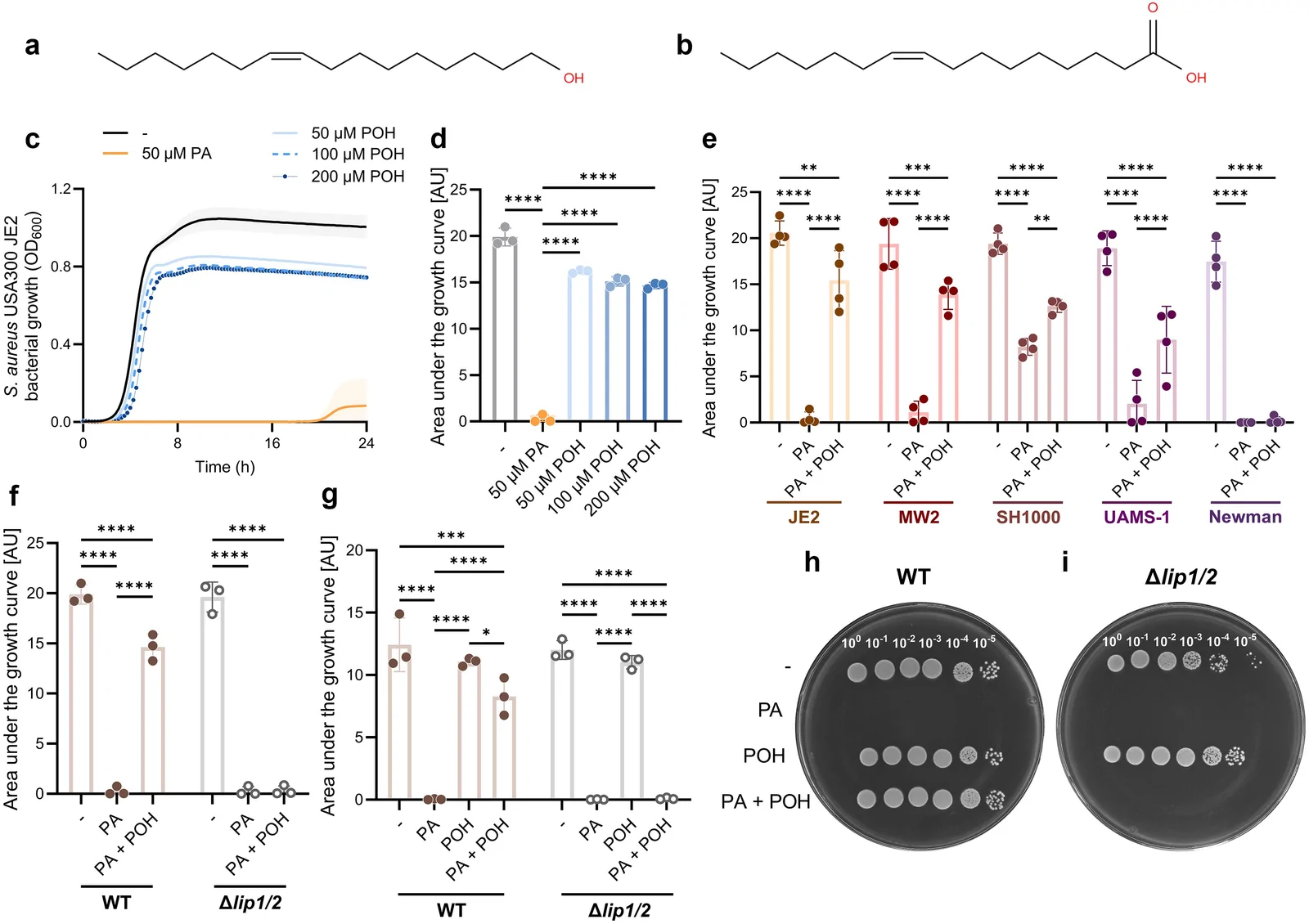
Fatty alcohols protect *S. aureus* from palmitoleic acid. (a–b) Molecular structure of palmitoleyl alcohol 16:1 (POH – a) or palmitoleic acid 16:1 (PA - b). (c–d) Optical density at 600 nm (OD_600_) was measured over 24 h to monitor the growth of the *S. aureus* USA300 JE2 in basic medium (BM) or BM supplemented with PA (50 μM) or POH (50, 100, or 200 μM). Growth curves (c) or computed area under growth curves using GraphPad Prism (d) are shown in arbitrary units (AU). (e) Area under growth curves of *S. aureus* strains USA300 JE2, USA400 MW2, SH1000, USA200 UAMS-1 and Newman grown in BM, BM supplemented with PA (50 μM), or PA and POH (50 μM) for 24 h. (f-i) Wild-type (WT) USA300 JE2 and its isogenic mutant defective for Lip1 and Lip2 (Δ*lip1/2*) were grown for 24 h in plain medium (BM for panel f, chemically defined medium (CDM) for panels g, h, and i), in medium supplemented with PA (50 μM) and/or POH (50 μM). Computed area under growth curves were plotted (f–g). WT and Δ*lip1/2* cultures in CDM were serially diluted and spotted onto TSA plates (h–i). A representative replicate from three independent experiments is presented. Data shown are means ± SD of 3 or 4 biological replicates. Statistical significance was determined by one-way ANOVA with Dunnett’s multiple comparisons test relative to the PA condition (d) or two-way ANOVA with Tukey’s multiple comparisons test (e-g). ^*^, *P* < .05; ^**^, *P* < .01; ^***^, *P* < .001; ^****^, *P* < .0001.

Recently, we have demonstrated that *S. aureus* utilises its lipase Lip2 to esterify and thereby detoxify AFAs with cholesterol [31]. Furthermore, we and others have shown that *S. aureus* generates ethyl esters from ethanol and AFAs [31, 41]. Since the increase in chain length of alcohols seemed to facilitate their *S. aureus*-mediated esterification with AFAs [31, 41], we surmised that long-chain fatty alcohols (FOH) would protect *S. aureus* against AFA toxicity. Drawing upon recent lipidomic profiling of human sebum, we estimate that physiological concentrations of free FOH are in the millimolar range [25, 42]. As representative FOH, we selected palmitoleyl alcohol (POH; Fig. 1a), the fatty alcohol analogue of palmitoleic acid (PA; Fig. 1b), a major AFA of mammalian skin [43] and human nasal fluid [44]. In agreement with prior research [45], we did not observe POH toxicity toward *S. aureus* (Fig. 1c, and  d), suggesting that the carboxyl group is required for AFA toxicity. However, the slightly extended lag phase, combined with the decreased OD_600_ at the stationary phase, suggests that *S. aureus* senses and responds to POH. To probe this, we monitored the expression of selected genes with documented (i) roles in AFA resistance (*farE*, *isdA*, and *lip2*), and/or (ii) responsiveness to AFA exposure (*clfA*, *clfB*, *fnbA*, *hla*, psmα, *saeR*) [31, 46–51]. In general, transcriptional changes induced by POH resemble those provoked by equimolar, subinhibitory concentrations of PA or the combination of PA and POH (Fig. S1). For instance, treatment with either PA or POH decreased *psmα* and *clfA* transcript levels (Fig. S1a). However, the two-fold induction of *farE* transcript levels by PA treatment was one order of magnitude lower than the POH-induced *farE* upregulation (Fig. S1b), suggesting that POH provokes distinct transcriptional changes in *S. aureus*. Because FarE mediates the efflux of AFAs [46, 50], the induction of *farE* expression by POH indicates that FOH regulate lipase-independent AFA resistance mechanisms. Next, we monitored the growth of various *S. aureus* strains in a rich medium, where all strains grew similarly. Except for SH1000, *S. aureus* strains USA300 JE2, USA400 MW2, USA200 UAMS-1, and Newman did not grow in the presence of 50 μM PA, ie, PA concentration in the nasal fluid [44] (Fig. 1e). In agreement with our previous observations with cholesterol [31], POH rescued the growth of all strains but Newman in the presence of otherwise inhibitory PA amounts (Fig. 1e). Since *lip2* (*geh*), the gene encoding for the lipase Lip2, is disrupted by the ΦNM4 prophage in Newman [52], these data suggested that POH-induced protection against PA was Lip2-dependent in *S. aureus*. Hence, we took advantage of the previously described and tested USA300 JE2 mutant [31, 53] defective for both Lip1 and Lip2 lipase production (Δ*lip1/2*). In clear contrast to its isogenic wild-type (WT) parental strain, Δ*lip1/2* was not protected by POH, neither in rich media (Fig. 1f and Fig. S2a) nor in a chemically defined medium [54] (CDM; Fig. 1g). In CDM, 50 μM POH was non-toxic, whereas 50 μM PA led to the eradication of *S. aureus* irrespective of lipase production (Fig. 1h-i). However, co-supplementation of PA and POH was innocuous to WT and fatal to Δ*lip1/2* (Fig. 1h-i). We also probed FOH-mediated protection from AFAs other than PA. SA (sapienic acid) is an AFA solely produced by human sebum [55], while LA (linoleic acid) is abundant throughout the human body [44]. The toxicity of SA or LA was alleviated by POH in a lipase-dependent manner (Fig. S2b).

We investigated the protective effect of FOH other than POH. Linoleyl alcohol (LOH), α-linolenyl alcohol (ALOH), or arachidonyl alcohol (AOH), did not alter the growth of *S. aureus* (Fig. S2c), but they all protected WT against PA. In contrast, Δ*lip1/2* remained vulnerable to PA despite FOH supplementation (Fig. S2d). Given that human skin is replete with saturated fatty acids, which, in contrast to unsaturated antimicrobial fatty acids (AFAs), are innocuous to *Staphylococcus aureus* [55], we sought to investigate whether saturated fatty acids would alter FOH-enabled AFA resistance. To probe this, we selected palmitic acid, the most abundant saturated fatty acid on the human skin surface [22, 30, 42, 56]. In contrast to its unsaturated counterpart, palmitoleic acid, palmitic acid did not abrogate the growth of *S. aureus* (Fig. S2e). Furthermore, palmitic acid supplementation did not inhibit the lipase-dependent protective role of palmitoleyl alcohol (POH) against palmitoleic acid (PA) (Fig. S2e). In 1:1 coculture assays, the lipase-expressing WT enabled the growth of its Δ*lip1/2* mutant in the presence of PA and POH (Fig. S3a-b). However, the competitive balance between WT and Δ*lip1/2* appeared slightly skewed toward WT (Fig. S3c), suggesting an advantage associated with lipase production. Taken together, our data illuminate the ability of *S. aureus* Lip2 and/or Lip1 to utilise FOH and enable bacterial survival and growth despite the presence of toxic AFAs.

### The lipase Lip2 is sufficient for FOH-elicited alleviation of AFA toxicity

**Figure 2:**
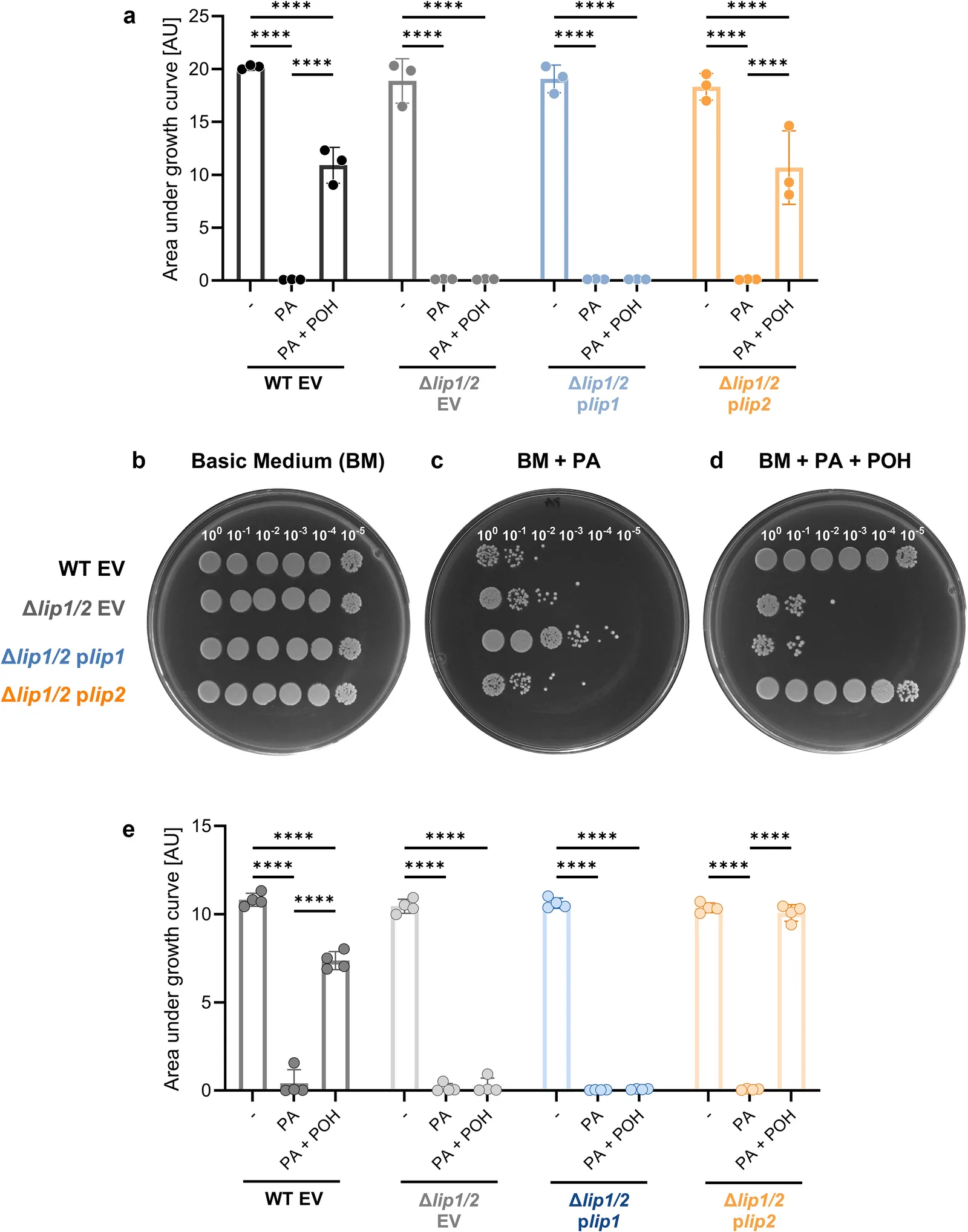
Lipase (Lip2) is required for fatty alcohol-mediated protection of *S. aureus* against AFAs. (a) Empty vector (EV)-bearing wild-type USA300 JE2 and its isogenic lipase-deficient mutant Δ*lip1/2*, or Δ*lip1/2* complemented with either p*lip1* or p*lip2* were grown for 24 h in basic medium (BM), or BM supplemented with 100 μM palmitoleic acid (PA), or 100 μM PA + 100 μM palmitoleyl alcohol (POH). Area under the growth curves in arbitrary units (AU) were computed. (b–d) Cultures of the bacteria as described in (a) in BM (b), BM + PA (c), or BM + POH (d) were serially diluted and spotted onto TSA plates. One representative biological replicate (*n* = 3) is shown. (e) Area under the growth curves of the strains detailed in (a) upon cultivation in chemically defined medium (CDM), CDM with PA (50 μM), or CDM with PA + POH (50 μM) for 24 h. Data shown are means ± SD of three or four biological replicates. Statistical significance was determined by two-way ANOVA with Tukey’s multiple comparisons test. ^****^, *P* < .0001.

The incapacity of Newman (a natural *lip2* mutant) to benefit from POH to grow in the presence of PA (Fig. 1e) strongly suggests that Lip2 is required for POH-mediated protection against PA. This is supported by our data obtained with the Δ*lip1/2* mutant (Fig. 1f, g, and  i). Next, we used Δ*lip1/2* mutants complemented with *lip1* or *lip2* on a plasmid (p*lip1* or p*lip2*, respectively) [31, 57]. The growth of the complemented strains in rich medium resembled that of the Δ*lip1/2* mutant carrying an empty vector (Fig. 2a-b). However, p*lip2*-complementation enabled the proliferation of Δ*lip1/2* in the presence of toxic amounts of PA and equimolar concentrations of POH, in rich medium (Fig. 2a-d), as well as in CDM (Fig. 2e). Together, these data demonstrate that Lip2 is necessary and sufficient for FOH-mediated protection from AFAs.

### Lipases from CoNS mediate FOH-aided resistance to AFAs

**Figure 3:**
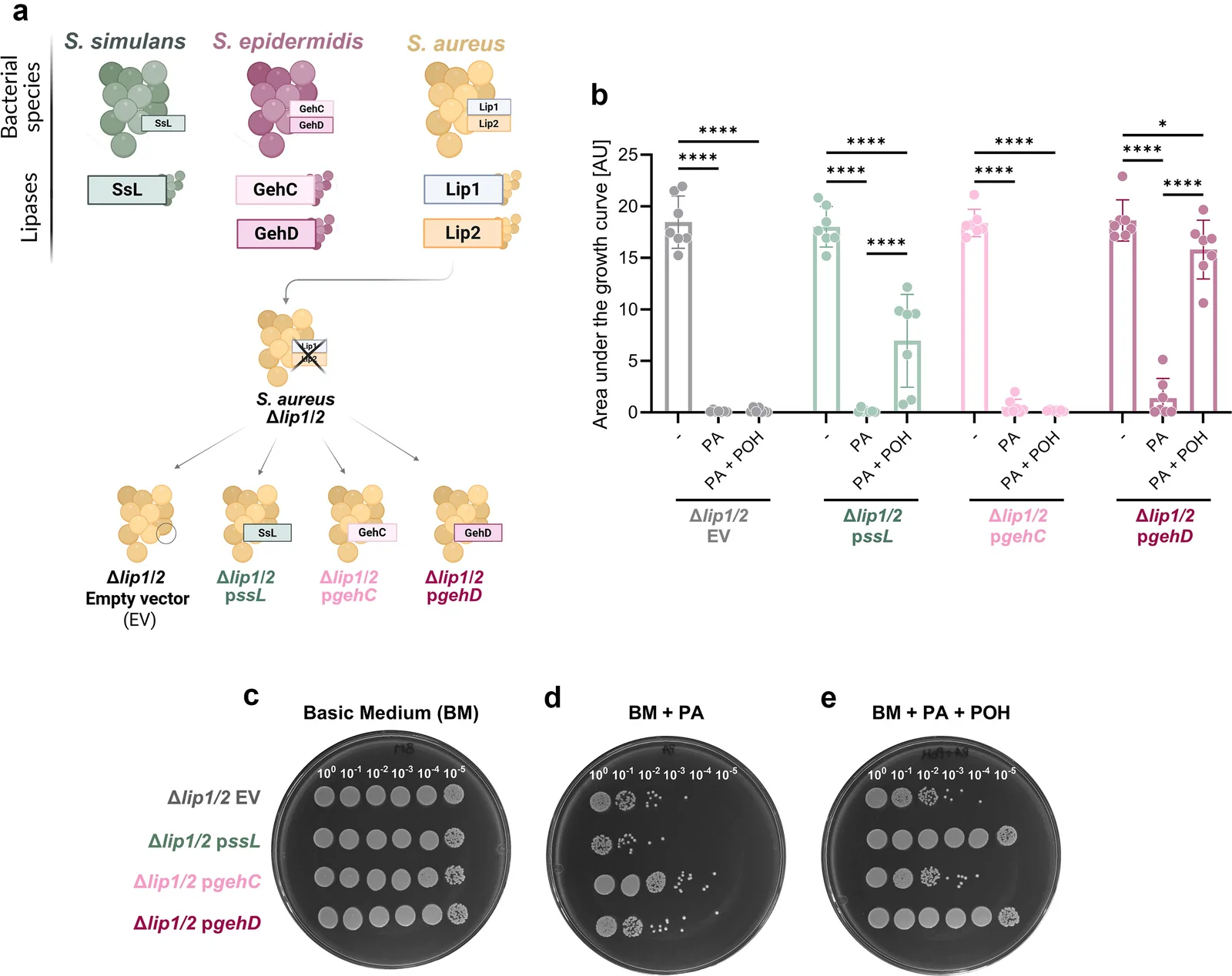
Lipases from coagulase-negative staphylococci enable fatty alcohol-aided AFA resistance. (a) Graphical depiction of the strategy used to generate a lipase-deficient *S. aureus* USA300 JE2 mutant (Δ*lip1/2*) heterologously expressing the lipases of *S. simulans* (SsL) or *S. epidermidis* (GehC or GehD). (b) Δ*lip1/2* carrying empty vector (EV) or Δ*lip1/2* complemented with p*ssL,* p*gehC*, or p*gehD* were grown for 24 h in basic medium (BM), BM supplemented with 100 μM PA, or BM supplemented with 100 μM PA + 100 μM POH. Area under the growth curves in arbitrary units (AU) were computed. (c–e) Cultures in BM (c), BM + PA (d), or BM + PA + POH (e) as described in (b) were serially diluted and spotted onto TSA plates. A representative biological replicate from seven independent experiments is presented. Data shown are means ± SD. Statistical significance was determined by two-way ANOVA with Tukey’s multiple comparisons test. ^*^, *P* < .05; ^**^, *P* < .01; ^****^, *P* < .0001.

Since *S. aureus* Δ*lip1/2* is unable to benefit from FOH to resist AFAs (Figs. 1 and 2), we reasoned that this mutant could be used as a chassis to express and probe lipases from coagulase-negative staphylococci (CoNS). Among CoNS, we selected *S. epidermidis* and *S. simulans* because (i) *S. epidermidis* is the most conspicuous staphylococcal competitor of *S. aureus*, and (ii) *S. simulans* appears to possess only one lipase with a secretion-directing signal peptide motif YSIRK [58], as revealed by manually inspecting the genome of *S. simulans* NCTC 11046 (ATCC 27848). This strain is a human skin isolate [59], which does not produce lysostaphin and was historically utilised to characterise the lysostaphin-producing organism as the *staphylolyticus* biovar of *S. simulans* [60]. The *S. simulans* lipase SsL, or a YSIRK lipase of *S. epidermidis* (GehC or GehD) was expressed on a plasmid in *S. aureus* Δ*lip1/2* (Fig. 3a). An amino acid sequence alignment revealed that GehC shares 85% sequence identity with Lip1, but only ~50% with Lip2, GehD and SsL, which share ~60% sequence identity with each other (Fig. S4a). SsL or GehD (not GehC) enabled POH-aided AFA resistance (Fig. 3b-e), which was mirrored by the lipase activity of the respective Δ*lip1/2*-conditioned media (Fig. S4b). These data suggest that the ability to promote FOH-dependent resistance to AFAs is a widespread feature of staphylococcal lipases.

**Figure 4:**
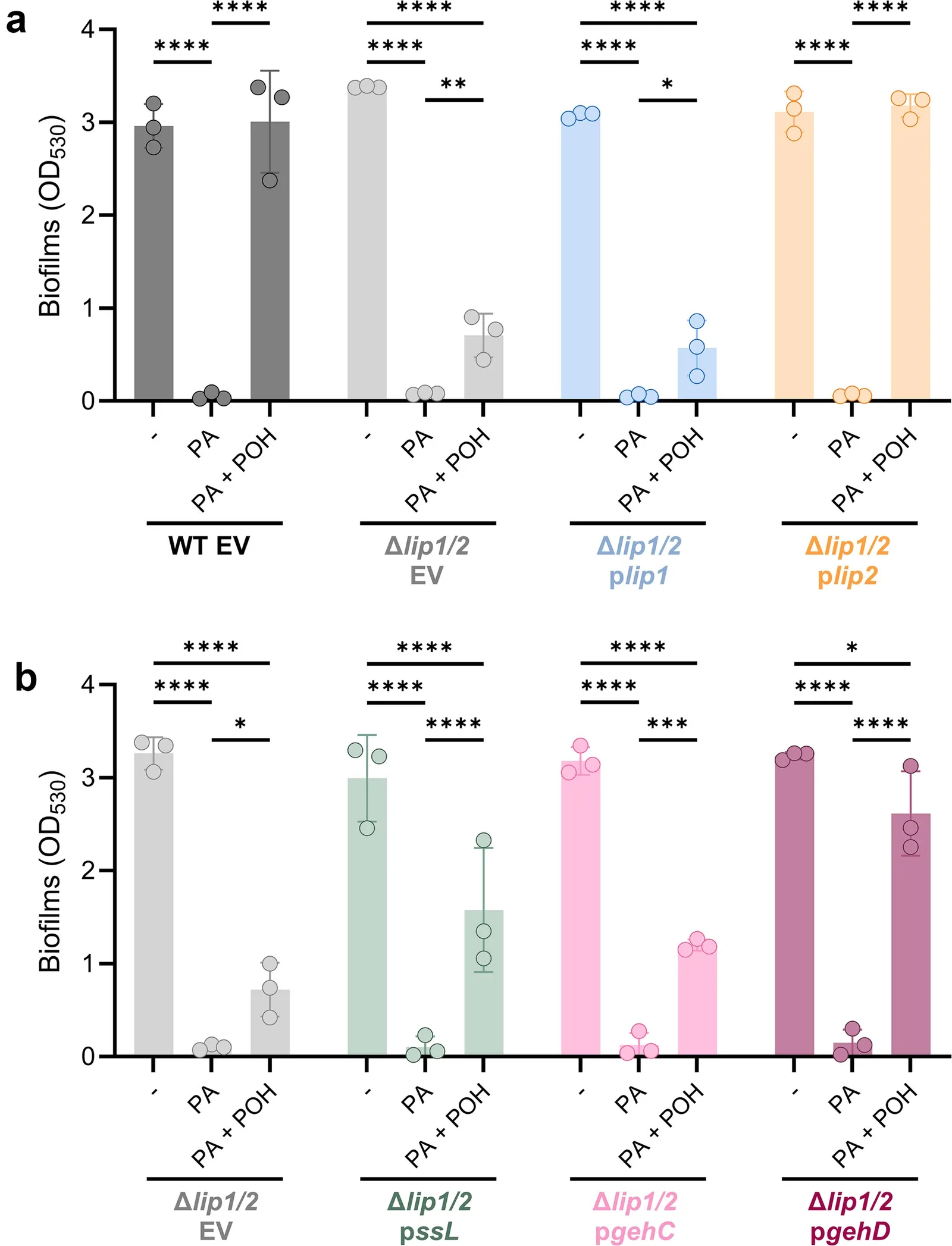
*Staphylococcus aureus* benefits from lipases and fatty alcohols to form biofilms despite the presence of AFAs. Wild-type USA300 JE2 and its isogenic Δ*lip1/2* mutant carrying empty vector (EV) or Δ*lip1/2* complemented with either p*lip1* or p*lip2* (a), or EV-bearing Δ*lip1/2* and Δ*lip1/2* complemented with p*ssL*, p*gehC*, or p*gehD* (b) were grown in basic medium (BM), BM supplemented with 100 μM palmitoleic acid (PA) or BM with 100 μM PA and 100 μM palmitoleyl alcohol (POH) for 24 h to form biofilms. Biofilms were stained with 0.1% safranin and optical density at 530 nm (OD_530_) was measured. Means ± SD of three biological replicates. Statistical significance was determined by two-way ANOVA with Tukey’s multiple comparisons test. ^*^, *P* < .05; ^**^, *P* < .01; ^***^, *P* < .001; ^****^, *P* < .0001.

Lipase-enabled, FOH-mediated AFA resistance was also investigated to assess active biofilm formation rather than the vulnerability of pre-existing biofilms. In rich medium, staphylococcal lipases appeared not to affect biofilm formation, which was prevented by 100 μM PA (Fig. 4a-b). Co-treatment with PA and POH enabled biofilm formation, which was further potentiated by Lip2 (Fig. 4a and Fig. S5a), SsL, GehC, and GehD (Fig. 4b and Fig. S5b). A lower PA concentration (75 μM) also prevented biofilm formation but revealed the capacity of Lip1 to boost AFA resistance in the presence of POH (Fig. S5c). We also investigated biofilm formation during treatment with POH alone. Irrespective of lipase expression, POH did not appear to affect *S. aureus* biofilms after a 24-h incubation (Fig. S6a). We surmised that any POH effect during the early stages of biofilm formation might be masked in these mature biofilms. Therefore, we halted the assay after 6 h to reassess the impact of POH. Indeed, POH enhanced the early establishment of biofilms by both WT *S. aureus* and the Δ*lip1/2* mutant (Fig. S6b). The effect of POH on *S. aureus* adherence during biofilm formation was further dissected by using an increased inoculum size. Under these conditions, Δ*lip1/2* formed more biofilm in response to POH than its wild type (Fig. S6c). This lipase-independent, POH-induced biofilm reshaping may protect *S. aureus* from AFAs. Collectively, these results suggest that staphylococcal lipases utilise FOH to alleviate AFA toxicity in both planktonic and biofilm settings.

### Staphylococcal lipases esterify FOH with AFAs to generate wax esters

**Figure 5:**
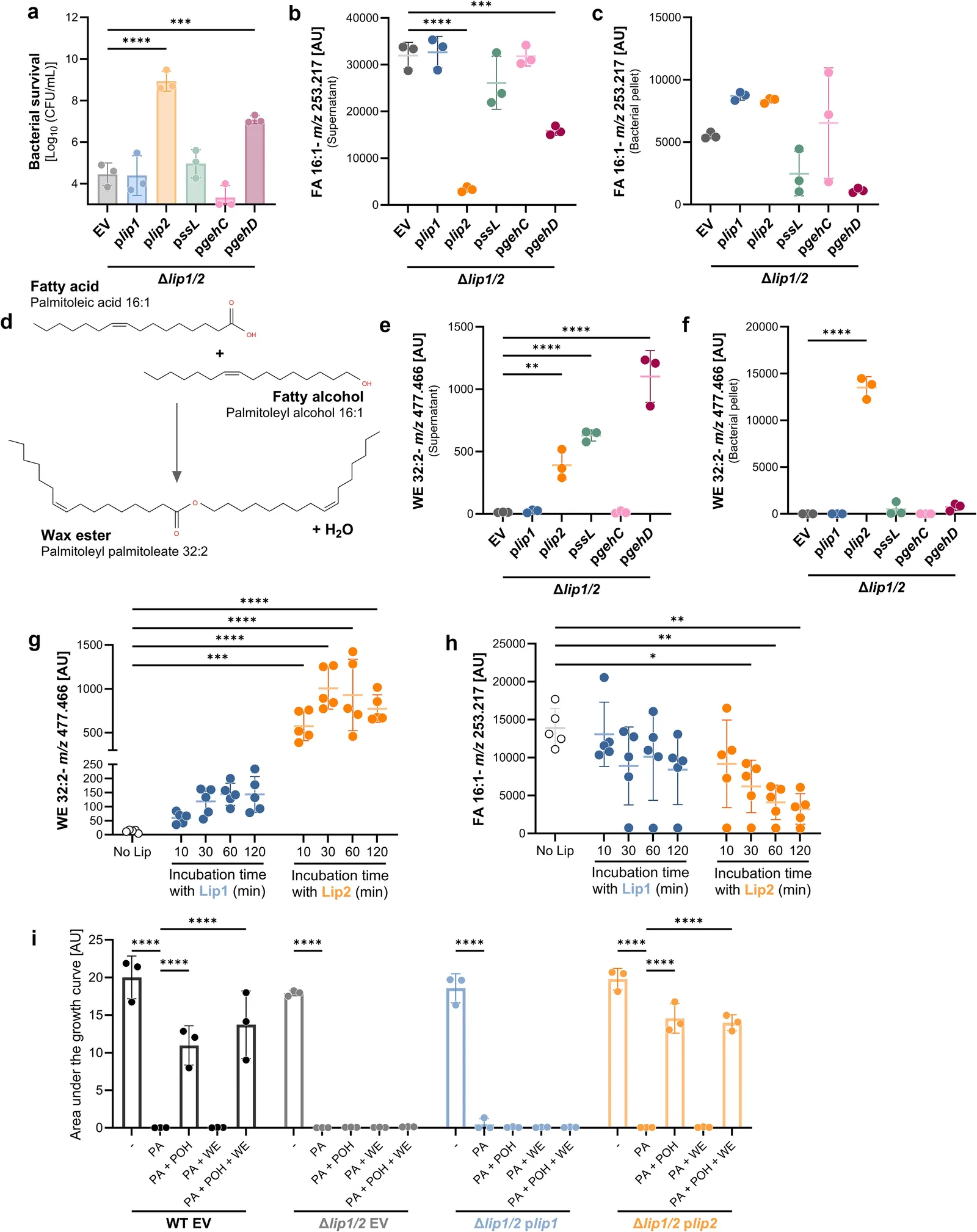
Staphylococcal lipases detoxify AFAs with fatty alcohols via wax ester production. (a) *Staphylococcus aureus* USA300 JE2 deficient for Lip1 and Lip2 (Δ*lip1/2*) carrying an empty vector (EV) or Δ*lip1/2* complemented with p*lip1*, p*lip2*, p*ssL*, p*gehC*, or *pgehD* were grown for 24 h in basic medium (BM) supplemented with 300 μM palmitoleic acid (PA) and 300 μM palmitoleyl alcohol (POH). After 24 h incubation, cultures were serially diluted and plated on TSA for CFU counting. (b–c) Supernatants and bacterial pellets of the cultures described in (a) were processed for lipidomic analysis via hydrophilic interaction liquid chromatography ion-mobility mass spectrometry (HILIC-IM-MS). Palmitoleic acid 16:1 (*m/z* 253.217) in negative ion mode ([M-H]^−^) from supernatants (b) and bacterial pellets (c) was measured. (d) Schematic representation of the lipase-catalysed esterification of palmitoleic acid and palmitoleyl alcohol leading to the production of wax ester (WE) palmitoleyl palmitoleate and water (H_2_O). (e–f) WE palmitoleyl palmitoleate 32:2 (*m/z* 477.4657) measurement using HILIC-IM-MS in positive mode ([M + H]^+^) from supernatants (e) and bacterial pellets (f) described in (a–c). (g–h) Recombinant *S. aureus* lipases Lip1 and Lip2 (1 μg/mL) were incubated for 10 to 120 min with 100 μM PA and 100 μM POH before lipid extraction and analysis using HILIC-IM-MS in positive and negative ion modes. Palmitoleyl palmitoleate WE 32:2 (*m/z* 477.466 – g) and palmitoleic acid FA (fatty acid) 16:1 (*m/z* 253.217–h) were measured. “No Lip” corresponds to 120 min incubation of PA and POH without recombinant lipase. (i) EV-bearing wild-type USA300 JE2 and Δ*lip1/2* or Lip1- or Lip2-proficient Δ*lip1/2* mutant (complemented with p*lip1* or p*lip2*, respectively) were cultivated in BM, BM supplemented with PA, PA, and POH, PA and palmitoleyl palmitoleate (WE), or PA + POH + WE (50 μM of each lipid) for 24 h. Area under the growth curves are shown in arbitrary units (AU). Data are means ± SD (*n* = 3 to 5). Statistical significance was determined by one-way ANOVA with Dunnett’s multiple comparisons test relative to the Δ*lip1/2* carrying EV (a–f) or the No Lip condition (g–h). Two-way ANOVA with Tukey’s comparisons test was applied to (i) for statistical analysis. ^*^, *P* < .05; ^**^, *P* < .01; ^***^, *P* < .001; ^****^, *P* < .0001.

In the presence of AFAs and protective FOH, the growth behavior of Δ*lip1/2* mutants expressing staphylococcal lipases agreed with their ability to hydrolyse ester substrates (Figs. 2 and 3 and Fig. S4b). Therefore, we performed growth assays in glass vials to analyze the lipid landscape associated with bacterial survival in the presence of PA and FOH. In agreement with assays in microtiter plates (Figs. 2 and 3), Δ*lip1/2* mutant complemented with either p*lip2* or p*gehD* was protected from PA toxicity in the presence of POH (Fig. 5a), and depleted free PA from its environment (Fig. 5b). However, differences in bacteria-associated free PA levels in lipase-complemented Δ*lip1/2*, as compared to the empty vector-bearing control, did not reach the threshold for statistical significance (Fig. 5c). We surmised that extracellular lipases used PA and POH for the biosynthesis of the wax ester (WE) palmitoleyl palmitoleate (Fig. 5d). WE was detected in the supernatants of Δ*lip1/2* with p*lip2*, *pssL* or p*gehD* (Fig. 5e) while only p*lip2* seemed to enable bacteria to accumulate WE (Fig. 5f). Moreover, the production of WE by lipases (Lip2, SsL or GehD), which boosted bacterial survival (Fig. 5a), was accompanied by increased phosphatidylglycerol levels in the corresponding strains (Fig. S7).

Next, we probed lipase-mediated WE synthesis in a cell-free system wherein recombinant lipases, AFAs, and FOH were mixed in phosphate buffer. WE and AFAs were measured upon incubation for various time points, followed by lipid extraction and lipidomic analysis via hydrophilic interaction liquid chromatography ion-mobility mass spectrometry (HILIC-IM-MS). Kinetics of WE synthesis revealed that Lip2 catalysed rapid and robust WE synthesis (Fig. 5g) leading to PA depletion (Fig. 5h). In contrast, WE detected in Lip1 samples after up to 120 minutes did not reach the threshold for statistical significance when compared to the control without recombinant lipase (Fig. 5g). However, after 24-h incubation, Lip1-mediated WE synthesis became apparent (Fig. S8a). This was also the case for Lip2, SsL and GehD. WE synthesis was concomitant with decline in PA levels (Fig. S8b). In addition to HILIC-IM-MS, we used HPTLC to easily monitor FOH during WE synthesis. Lipase-catalysed WE synthesis led to concomitant decline in FOH and AFAs (Fig. S8c–h). More specifically, POH and PA were used by Lip1, Lip2, SsL, or GehD for WE synthesis (Fig. S8c). In addition to POH, we also tested other FOH with PA and Lip2. WE were detected with all tested FOH (Fig. S8d). Using POH, we also investigated the substrate preference of Lip1 or Lip2 for various AFAs. Subtle differences in WE synthesis between Lip1 and Lip2 were noticeable when monounsaturated oleic acid was tested (Fig. S8e). These differences were exacerbated with polyunsaturated linoleic acid (Fig. S8e), α-linolenic acid, and arachidonic acid (Fig. S8f). Lip2 was more apt than Lip1 at using AFAs for WE synthesis (Fig. S8e and f). Importantly, the catalytically inactive Lip2^S412A^ was unable to detoxify AFAs via esterification with FOH (Fig. S8g and h).

Besides WE synthesis, we investigated whether recombinant staphylococcal lipases could hydrolyse WE by mixing and incubating these lipases with WE. To a variable extent, all lipases tested (Lip1, Lip2, SsL, GehD, and even GehC) released PA and POH from WE at pH 6 (Fig. S9a), pH 7 (Fig. S9b), or pH 8 (Fig. S9c). Since lipases can synthesise and hydrolyse WE, we investigated how PA-treated bacteria would grow in the presence of POH and/or WE. Clearly, Lip2-mediated AFA resistance induced by POH was unaffected by WE supplementation in rich medium (Fig. 5i). In CDM, WE alone did not impact the growth of *S. aureus*, irrespective of Lip1 and/or Lip2 proficiency (Fig. S9d). Like in rich medium, WE did not prevent protection from PA by POH in CDM (Fig. S9e). Taken together, our results strongly suggest that staphylococcal lipases exhibit differential abilities to synthesise or hydrolyse WE. However, lipase-mediated WE synthesis must take place at a high rate to allow AFA detoxification and bacterial growth.

### FOH-enabled interspecies collective resistance to AFAs

**Figure 6:**
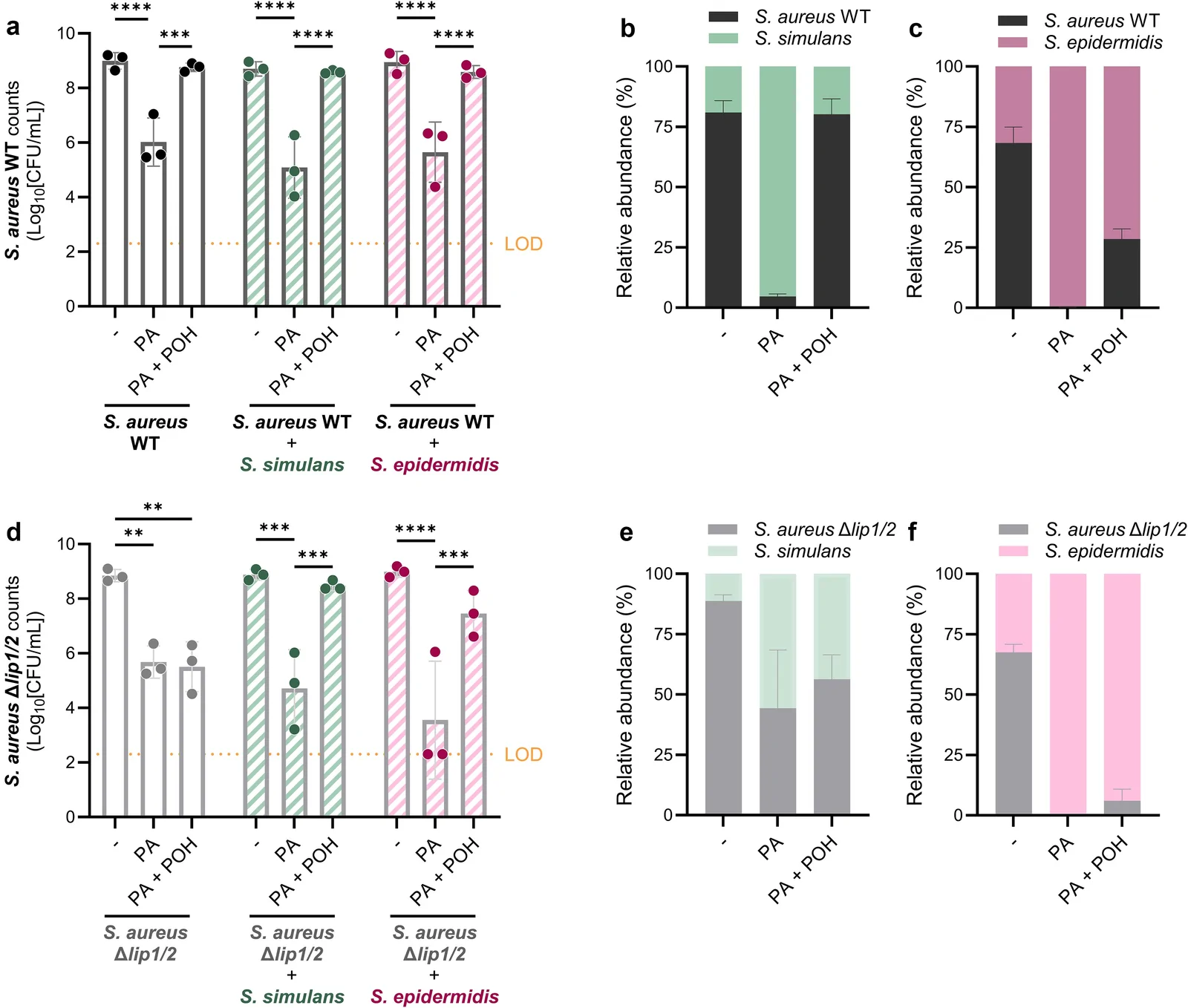
Interspecies collective AFA resistance is enabled by fatty alcohols. Wild-type (WT) *S. aureus* USA300 JE2 (a–c) or its isogenic Δ*lip1/2* mutant (d–f) was cultured alone or co-cultured with coagulase-negative staphylococci *S. simulans* or *S. epidermidis* in basic medium (BM), BM with palmitoleic acid (PA–200 μM), or BM with PA and palmitoleyl alcohol (POH—200 μM). After 24 h incubation, cultures were serially diluted and selectively plated for colony-forming unit (CFU) counting. CFU counts (a or d) were used to calculate the relative abundance of *S. aureus* (WT or Δ*lip1/2*) and *S. simulans* (b or e) or *S. epidermidis* (c or f). Data shown are means ± SD of three biological replicates. Statistical significance was determined by two-way ANOVA with Tukey’s multiple comparisons test (a and d). ^**^, *P* < .01; ^***^, *P* < .001; ^****^, *P* < .0001. LOD indicates the limit of detection [log_10_(203 CFU/ml) = 2.3].

Thus far, we have utilised heterologous expression in *S. aureus* Δ*lip1/2* or recombinant proteins to demonstrate that lipases of CoNS metabolize WE. Next, we sought to investigate these lipases within the context of parental CoNS strains. Rich medium conditioned by *S. simulans* or *S. epidermidis* displayed lipase activity, as assessed with fatty acid ester substrates (Fig. S10a and b). Similar to WT *S. aureus* (Fig. 6a), lipase-expressing *S. simulans* or *S. epidermidis* is able to benefit from POH to resist PA in pure cultures (Fig. S10c and d). Since *S. epidermidis* was several orders of magnitude more resistant to PA than *S. aureus*, we assessed the competitive fitness of *S. aureus* in a 1:1 coculture with CoNS during exposure to PA alone, or PA combined with POH. After 24 h of growth in basic medium, *S. aureus* dominated *S. simulans* (Fig. 6b) or *S. epidermidis* (Fig. 6c). Given that *S. epidermidis* reached CFU counts approximately six-fold higher in pure culture than when cocultured with *S. aureus* in basic medium (Fig. S10d), the dominance of *S. aureus* in these cocultures likely reflects interspecies competition. By contrast, *S. simulans* remained largely unaffected by *S. aureus* in basic medium, despite exhibiting a reduced baseline growth capacity (Fig. S10c). However, PA supplementation enabled these CoNS to outcompete *S. aureus* (Fig. 6b and c). PA detoxification with POH restored the dominance of *S. aureus* over *S. simulans* (Fig. 6b) but only decreased the relative abundance of *S. epidermidis* from ~100% to ~72% (Fig. 6c). While the lipase-deficient Δ*lip1/2 S. aureus* mutant was unable to utilise POH to resist PA in pure cultures (Fig. 6d), its growth in cocultures with CoNS increased by several orders of magnitude when both PA and POH were present compared to PA alone (Fig. 6d). Nevertheless, this enhanced survival did not prevent the PA-induced loss of Δ*lip1/2* dominance over *S. simulans* (Fig. 6e) or *S. epidermidis* (Fig. 6f). Collectively, our data suggest that skin CoNS outcompete *S. aureus* in AFA-rich environments such as the skin. However, AFA detoxification with FOH by CoNS enables the growth of otherwise AFA-susceptible *S. aureus*. A phenomenon that we termed interspecies collective AFA resistance.

### Lipase is required for FOH-mediated interspecies collective AFA resistance

Since interspecies collective AFA resistance was promoted by FOH, we surmised that lipases were required to esterify and thereby detoxify AFAs with FOH. To test this, we took on the challenge of generating the first knockout mutant in a *S. simulans* background. As detailed in Methods, we utilised a Φ187-transduced pIMAY construct to genetically manipulate WT *S. simulans* and generate a lipase SsL-deficient mutant. This Δ*ssL* mutant, in stark contrast to its parental WT, displayed almost undetectable lipase activity irrespective of the fatty acid substrate used (Fig. S10a), suggesting that SsL is the major if not the unique lipase of *S. simulans*.

**Figure 7:**
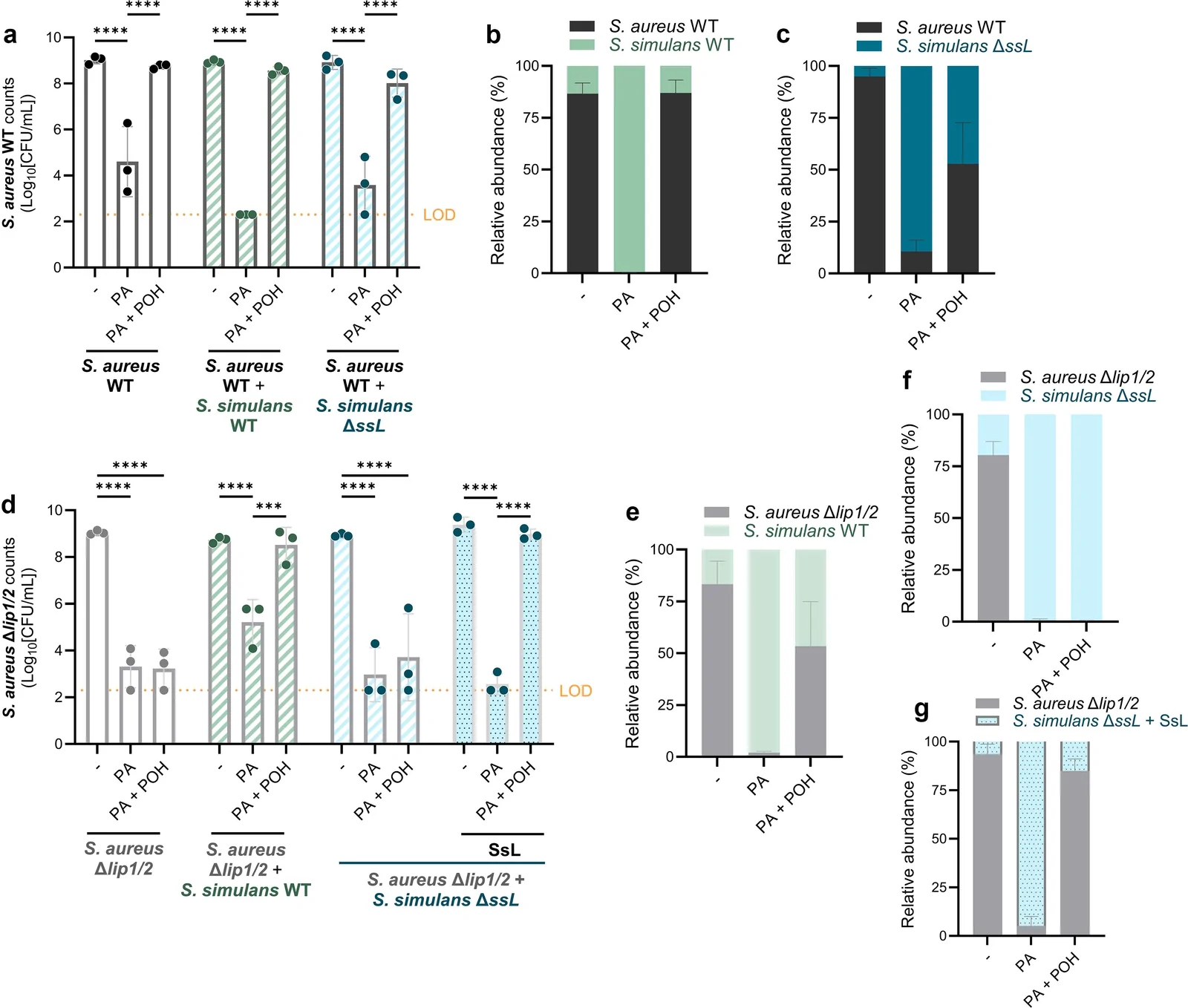
Fatty alcohol-mediated interspecies collective AFA resistance is lipase-dependent. (a–c) Wild-type (WT) *S. aureus* USA300 JE2 was cultured alone or co-cultured with WT *S. simulans* or its SsL-deficient mutant *(S. simulans* Δ*ssL*) in basic medium (BM), BM with 200 μM palmitoleic acid (PA), or BM with 200 μM PA and 200 μM palmitoleyl alcohol (POH) for 24 h. Cultures were then serially diluted for selective plating and colony-forming unit (CFU) enumeration. Plotted are CFU counts (a) as well as relative abundance of WT *S. aureus* and WT *S. simulans* (b) or *S. simulans* Δ*ssL* (c). (d–g) *S. aureus* Δ*lip1/2* was cultured alone or co-cultured with WT *S. simulans*, *S. simulans* Δ*ssL*, or *S. simulans* Δ*ssL* supplemented with recombinant SsL (1 ng/μL) as described in a–c. CFU counts (d) were used to calculate the relative abundance of *S. aureus* Δ*lip1/2* and WT *S. simulans* (e), *S. simulans* Δ*ssL* (f), or *S. simulans* Δ*ssL* + recombinant SsL (g). LOD indicates the limit of detection [log_10_(203 CFU/ml) = 2.3]. Data shown are means ± SD of three biological replicates. Statistical significance was determined by two-way ANOVA with Tukey’s multiple comparisons test. ^***^, *P* < .001; ^****^, *P* < .0001.

Inhibition of WT *S. aureus* growth by PA was alleviated by POH in pure culture or cocultures with Δ*ssL* or its WT (Fig. 7a). While WT *S. simulans* was outcompeted by WT *S. aureus* upon PA detoxification with POH (Figs 6b and 7b), Δ*ssL* remarkably maintained competitive parity with WT *S. aureus* (Fig. 7c). Unlike its WT, Δ*ssL* failed to protect Δ*lip1/2* from PA toxicity in the presence of POH (Fig. 7d and S11a). Consequently, while WT *S. simulans* and Δ*lip1/2* maintained near parity under PA and POH treatment (Figs 6e, 7e, and  S11b), Δ*ssL* completely dominated Δ*lip1/2* under identical conditions (Figs 7f and S11c). Intriguingly, WT *S. simulans* and Δ*ssL* were protected from PA by POH in monocultures or cocultures (Fig. S11d–g). Lipase-independent POH-triggered resistance to PA suggests transcriptional reprogramming in response to POH.

To exclude the metabolic burden of SsL lipase production as a confounding factor, we supplemented the coculture of lipase-deficient *S. aureus* Δ*lip1/2* and *S. simulans* Δ*ssL* with recombinant SsL. This led to near eradication of Δ*lip1/2* by PA alone, and outgrowth of Δ*lip1/2* in the presence of PA and POH (Fig. 7d and g). Taken together, our data indicate that lipase-mediated detoxification of AFAs with FOH enables the growth of lipase-deficient members of microbial consortia.

### Collective AFA resistance within mixed biofilms

To determine whether collective AFA resistance manifests also within biofilms, *S. aureus* (WT or Δ*lip1/2*) was incubated under static conditions to form biofilm in monoculture or in 1:1 coculture with CoNS. Viable bacteria within biofilms were enumerated to assess the impact of PA treatments in rich medium. As with planktonic cultures, within biofilms, POH protected WT *S. aureus* from PA in monocultures or cocultures with *S. simulans* or *S. epidermidis* (Fig. S12a). PA-induced dominance of CoNS was abolished by POH (Fig. S12b and c), which also promoted the growth of *S. simulans* (Fig. S12d). *Staphylococcus epidermidis* resisted PA regardless of POH (Fig. S12e). *Staphylococcus aureus* Δ*lip1/2* biofilms exhibited POH-induced protection from PA (Fig. S12f). However, in coculture with *S. simulans*, Δ*lip1/2* grew four orders of magnitude more with PA and POH as compared to PA alone (Fig. S12f). This was mirrored by the relative abundance of Δ*lip1/2* compared to *S. simulans* (Fig. S12g). In contrast, in coculture with *S. epidermidis*, the Δ*lip1/2* mutant did not experience the same level of protection (Fig. S12f and h), suggesting localised or absent POH-mediated PA detoxification.

We investigated viable staphylococci within CDM biofilms. For WT *S. aureus*, although POH-mediated protection was apparent in CDM monocultures or cocultures (~2-log increase) (Fig. S13a), it fell short of the 4-log protection observed in rich medium (Fig. S12a). However, increased PA toxicity toward *S. simulans* in CDM (Fig. S13b) resulted in near competitive parity between *S. simulans* and WT *S. aureus* (Fig. S13c). Both bacterial species were two to four orders of magnitude more susceptible to PA than *S. epidermidis* (Fig. S13d), which outcompeted WT *S. aureus* when PA was present (Fig. S13e). CDM biofilms of Δ*lip1/2* mutant (Fig. S13f–h) strongly phenocopied biofilms in rich medium (Fig. S12F–h). Together, our biofilm data illuminate POH-mediated detoxification of PA, and not just mere resistance to PA, as a prerequisite for interspecies collective AFA resistance.

### Salt alleviates AFA toxicity and AFA-enabled dominance of CoNS over *S. aureus*

**Figure 8:**
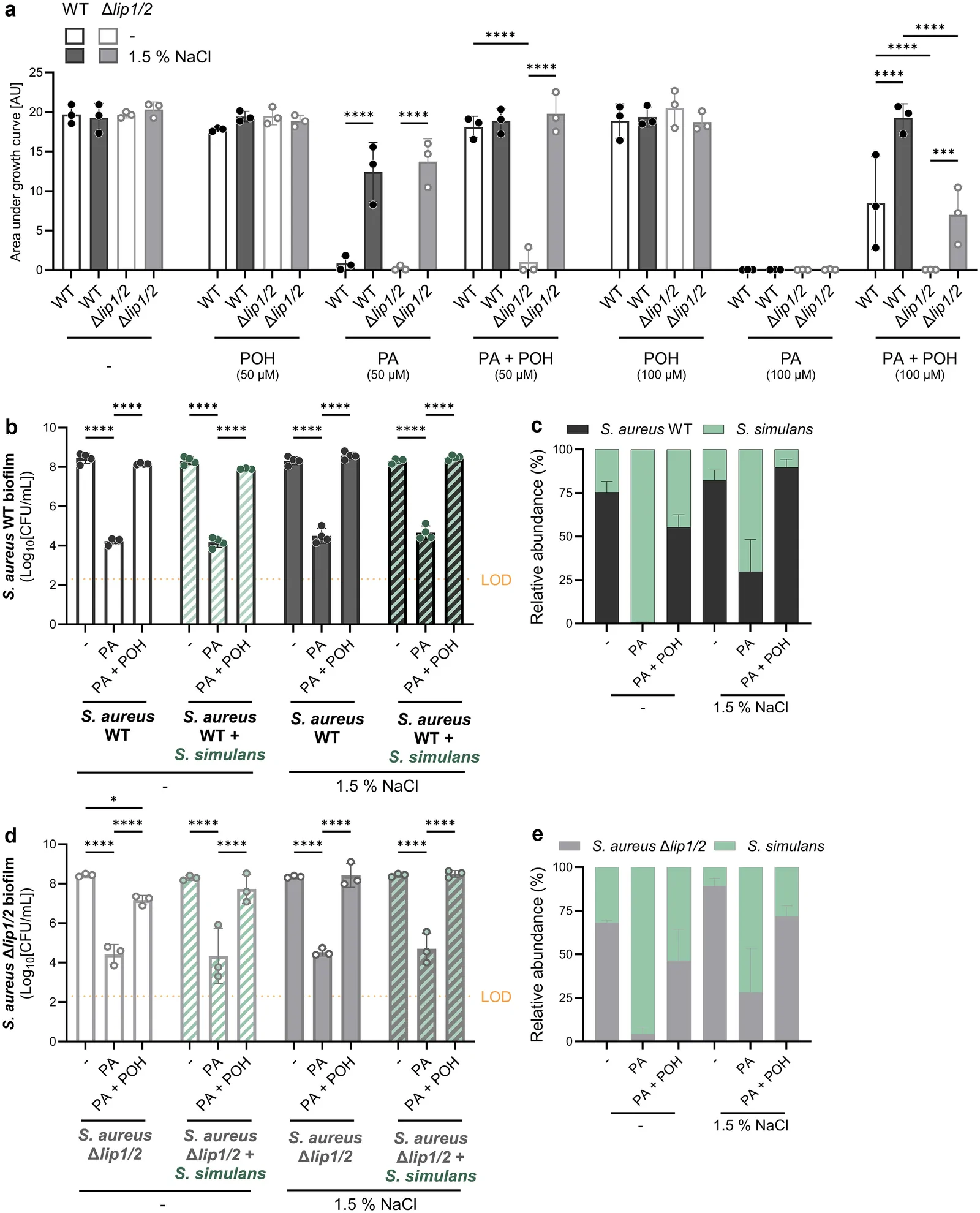
Salt stress alleviates AFA toxicity toward *S. aureus*. (a) Wild-type USA300 JE2 and its isogenic lipase-deficient mutant Δ*lip1/2* were grown for 24 h in plain medium [basic medium (BM) or BM supplemented with 1.5% NaCl], or medium supplemented palmitoleic acid (PA–50 or 100 μM), or equimolar concentrations of PA and palmitoleyl alcohol (POH–50 or 100 μM). Area under the growth curves in arbitrary units (AU) were computed. (b–e) WT *S. aureus* or Δ*lip1/2* mutant was cultured alone or co-cultured with *S. simulans* for 24 h to form biofilms. Bacteria were grown in plain medium (BM or BM supplemented with 1.5% NaCl), or medium supplemented with 100 μM PA or 100 μM PA and 100 μM POH. Biofilms were mechanically disrupted, serially diluted and selectively plated for colony-forming unit (CFU) enumeration. CFU counts (b and d) were used to calculate the relative abundance of *S. aureus* (WT–c or Δ*lip1/2*–e) and *S. simulans*. LOD indicates the limit of detection [log_10_(203 CFU/mL) = 2.3]. Data shown are means ± SD of three or four biological replicates. Statistical significance was determined by two-way ANOVA with Tukey’s multiple comparisons test. ^*^, *P* < .05; ^***^, *P* < .001; ^****^, *P* < .0001.

Lipase-mediated esterification of PA with POH generates not only WE but also water (Fig. 5d). Therefore, we surmised that WE synthesis may be exploited by staphylococci to resist desiccation and/or high-salt stress, which are pervasive on skin [7]. To investigate this, we monitored the growth of WT *S. aureus* and its Δ*lip1/2* mutant individually in rich medium supplemented with 1.5% (15 g/L) NaCl. Under these high-salt conditions, PA toxicity toward *S. aureus* decreased and a lipase-independent, POH-triggered PA resistance emerged (Fig. 8a). Conversely, the AFA resistance of CoNS during high-salt stress remained either unchanged (*S. epidermidis*, Fig. S14a) or decreased (*S. simulans*, Fig. s14b). Consistent with its increased AFA resistance (Fig. 8a-b), WT *S. aureus* resisted complete dominance by *S. simulans* in biofilms exposed to PA and elevated salt (Fig. 8c). In high-salt biofilms, the *S. aureus* Δ*lip1/2* mutant was so remarkable at resisting PA in the presence of POH (Fig. 8d) that it outcompeted *S. simulans* under these conditions (Fig. 8e). Unlike *S. aureus* (WT or Δ*lip1/2*), *S. simulans* exhibited a 1-log increase in PA susceptibility during salt stress (Fig. S14c). This heightened PA toxicity was nullified by POH, but not by coculture with *S. aureus* (Fig. S14c), suggesting that salt-induced AFA resistance functions as a private good restricted to *S. aureus*.

High salt did not alter the AFA resistance of *S. epidermidis* in pure or mixed biofilms with *S. aureus* (WT or Δ*lip1/2*) (Fig. S15a). Within these biofilms, WT *S. aureus* utilised POH to resist PA (Fig. s15b) so effectively that it dominated *S. epidermidis* (Fig. S15c). Conversely, Δ*lip1/2* exhibited enhanced AFA resistance in the presence of POH and high salt (Fig. S15d) but failed to outcompete *S. epidermidis* under these conditions irrespective of POH supplementation (Fig. S15e).

We utilised high salt conditions to investigate the capacity of *S. aureus* (WT or Δ*lip1/2*) to invade *S. simulans* (WT or Δ*ssL*) preformed biofilms. These invasion assays were conducted concurrently with treatment with PA alone or a PA/POH combination. In general, *S. aureus* was highly adept at invading preformed *S. simulans* biofilms (Fig. S16a and b). Pre-established *S. simulans* biofilms enabled *S. aureus* to reach ~10^6^ CFU (Fig. S16a and b) despite PA treatment, in contrast to the ~10^4^ CFU enumerated in pure biofilms (Fig. 8b and d). Despite this relatively high survival rate during PA treatment, a subtle, but clear POH- and lipase-dependent protection from PA was apparent (Fig. S16a and b). When preformed *S. simulans* biofilms were treated with PA in the absence of invading *S. aureus*, the biofilms were not destroyed (Fig. S16c and d). However, PA mildly but consistently inhibited *S. simulans* growth within biofilms, allowing the protective properties of POH to become apparent for WT *S. simulans* (Fig. S16c), but not for Δ*ssL* (Fig. S16d). Despite the vulnerability of *S. simulans* biofilms to invasion, *S. simulans* maintained its overall dominance over *S. aureus* regardless of lipase expression or lipid treatment (Fig. S16e–h). However, this dominance was markedly exacerbated by PA exposure. The addition of POH mildly attenuated this PA-driven exacerbation, an effect observed in the presence of the lipase-expressing WT *S. aureus*, but not the Δ*lip1/2* mutant (Fig. S16e–h). Taken together, our data unveil how lipase-proficient, WE-producing *S. aureus* uniquely leverages elevated salt to survive CoNS competition in skin-mimicking conditions.

## Discussion

The skin surface is a mosaic of highly diverse microenvironments that are generally lipid-rich, acidic, and high in salt. Within these distinct niches, microbe–microbe and microbe–host interactions determine the balance between health and pathology. Since lipids serve as cues or weapons during these interactions, recent research has pivoted toward characterising how an altered lipid landscape drives skin dysbiosis [8, 23–27]. While the correlation between dysbiosis and various skin disorders is well-documented, the extent to which microbial lipid metabolism actively dictates disease progression remains poorly defined. Because lipidomic analyses only capture static snapshots of the skin environment, understanding disease dynamics requires longitudinal studies and a detailed mechanistic understanding of how microbes alter host lipids. To address the latter, here we have investigated how staphylococcal lipases contribute to wax ester pools. We demonstrate that staphylococcal lipases catalyse the hydrolysis and synthesis of WE. Lipase-mediated WE synthesis detoxifies AFAs by esterification with FOH in planktonic and biofilm settings. Furthermore, we show that WE production drives interspecies collective AFA resistance, whereby a lipase-deficient AFA-susceptible species benefits from AFA detoxification by a lipase-proficient co-cultivated species. Thus, we uncovered a previously unidentified mechanism by which bacteria metabolise wax esters with potential implications for the skin barrier and microbiome.

A limitation of our study is that the mechanistic insights are derived from in vitro assays. However, several lines of evidence suggest that our findings could translate to the native skin environment. First, using the *S. aureus* USA300 JE2 strain pair (wild type and Δ*lip1/2* mutant), we have previously demonstrated that bacterial proliferation on mouse skin occurs in a lipid- and lipase-dependent manner [31]. Second, *S. aureus* utilises its lipases (primarily Lip2) to invade hair follicles following topical application to the mouse skin [61]. Third, *lip2*/*geh* transcripts are detectable on atopic dermatitis skin [62] and upregulated in *S. aureus* during proliferation on healthy human skin explants [63]. By contrast, there is no direct evidence that *S. epidermidis* GehC and/or GehD are produced on human skin. However, recent skin metatranscriptomic datasets contain features annotated as triacylglycerol lipases, though they were not explicitly detailed as *gehC* or *gehD* transcripts [64]. These are considered core genes for skin-colonising staphylococci (*gehC*) and are predicted to be involved in species-specific niche occupancy (*gehD*) [65]. The functional importance of these cutaneous staphylococcal lipases is supported by a landmark study demonstrating that, in the absence of *C. acnes*, coagulase-negative *staphylococci* are likely responsible for releasing 50% to 100% of the free fatty acids from sebum esters [66]. These free fatty acids account for approximately one-fifth of the skin surface lipid film, reaching hundreds of millimolar to molar concentrations similar to those of wax esters or triacylglycerols [7, 30]. Given that our in vitro assays utilised conservative micromolar concentrations of lipase substrates, the wax ester metabolism detailed in this study likely takes place natively on the skin.

Beyond lipase-mediated esterification into cholesteryl esters or wax esters, *S. aureus* has evolved several mechanisms to counteract antimicrobial fatty acids (AFAs) [31, 43, 46, 47, 49, 50, 67]. For instance, hydrophilic cell-wall anchored glycopolymers (wall teichoic acids) [68–70] and proteins (IsdA) [47] hinder the interaction of hydrophobic AFAs with bacterial cells. Furthermore, the efflux pump FarE prevents the accumulation of cell-associated AFAs [46, 50]. Additionally, the oleate hydratase OhyA converts, in vitro and in vivo, AFAs containing a *cis*-9 double bond into hydroxy fatty acids with immunomodulatory properties [43, 71, 72]. The functional importance of OhyA and FarE extends to vaginal lactobacilli. These proteins are absent in vaginosis-associated, AFA-susceptible *Lactobacillus iners*, yet are expressed by the health-promoting *L. crispatus*, suggesting a potential for AFA-based therapeutics against bacterial vaginosis [73].

Our findings align with cumulative evidence that skin-dwelling CoNS, such as *S. epidermidis*, exhibit greater resistance to AFAs compared to *S. aureus* [74, 75]. We demonstrate that AFA supplementation shifts the competitive balance, allowing *S. epidermidis* to dominate in media that otherwise support *S. aureus* outgrowth. A similar ecological precedent exists on the nasal epithelium, where *S. epidermidis* dominance over *S. aureus* is facilitated by host-derived defenses, specifically antimicrobial peptides [76]. Exceptional AFA resistance likely explains why *S. epidermidis* grows more robustly than *S. aureus* in skin-like media containing high concentrations of artificial sebum and sweat [77]. Although the mechanisms driving this unique resistance remain unknown, this phenotype highlights that adaptation to the AFA-rich skin environment is a prerequisite for successful colonisation. Consistent with this, we reveal that skin-specific cues, such as fatty alcohols or salt, enhance AFA resistance in a lipase-independent manner. In cocultures, these resistance mechanisms are species-specific, strongly suggesting the involvement of cell wall or membrane remodeling rather than secreted factors [46, 47, 49, 67, 69]. From an ecological perspective, invading pathogens are unlikely to benefit from the detoxification of the skin by resident commensals such as *S. epidermidis*. This bacterium resists AFAs, produces WEs, and successfully outcompetes *S. aureus* in AFA-rich conditions. This suggests that *S. epidermidis* may sequester its lipases at the cell surface, as previously demonstrated for *S. aureus* [78, 79], to detoxify its immediate microenvironment, while leaving the surrounding macro-environment inhospitable to invading microbes.

Microbes can manipulate skin barrier/repair in many ways. For instance, *C. acnes* [20] and *R. mucosa* [21] induce skin lipid synthesis. *S. epidermidis* employs its sphingomyelinase Sph to produce ceramides, major lipids required for skin barrier integrity [17]. With WE, we may have uncovered yet another lipid class that is targeted by microbes to impair or maintain skin integrity. In keeping with this, mice with atrophic sebaceous glands and decreased WE production display heightened susceptibility to bacterial infections [80, 81].

WE have previously been reported to be produced by bacteria and archaea as intracellular storage lipids [82–85]. In these organisms, WE are synthesized intracellularly by a wax ester synthase/acyl-coenzyme A:diacylglycerol acyltransferase, which catalyzes the esterification of FOH with fatty acyl-coenzyme A. In contrast, we reveal extracellular WE production mediated by staphylococcal lipases in a coenzyme-A-independent manner. In addition to CoNS, other skin microorganisms secrete potent lipases, including *C. acnes* [19], *C. accolens [**15**]*, and *Malassezia* spp. [86]. This raises the possibility that WE metabolism represents a widespread and previously underappreciated activity of microbial lipases.

Lipases possess both hydrolytic and synthetic (esterification) capabilities, as we demonstrate here through both WE synthesis and degradation. Notably, the extent of WE hydrolysis varies depending on the environmental pH and the specific staphylococcal lipase employed. It is tempting to hypothesise that the aggregate lipase activity of the microbiome dictates whether overall WE levels remain stable or undergo dramatic alteration. While WE levels appear elevated on the skin surface of young men with acne [28], the extent to which *C. acnes* or other microbes drive this phenomenon remains unclear. Conversely, WE have not yet been implicated in AD; however, impaired sebum production has been documented in AD patients, with decreased triacylglycerol levels correlating with disease severity [27]. Consistent with this observation, sebum-lacking mice develop AD-like dermatitis [87]. Furthermore, in lesional AD skin, elevated salt acts as an ionic checkpoint for T_H_2-driven chronic inflammation [88]. Since our data demonstrate an increased AFA resistance of *S. aureus* under high-salt stress, they provide an additional explanation as to why this bacterium proliferates in lipid-depleted AD skin. Therefore, restoring the lipid landscape through the application of WE-synthesizing CoNS or WE supplementation may represent a therapeutic avenue for skin disorders characterised by impaired sebaceous function.

## Conclusion

Collectively, our study illuminates WE as both products and substrates of staphylococcal lipases, which likely play an underestimated role in shaping the skin lipid landscape and microbiome. Moreover, this work lays the foundation for future investigations into the impact of WE on skin disorders.

## Supplementary Material

WE_paper_Supplementary_files_R2_wrag148

## Data Availability

Lipidomics data are available on the MassIVE repository under accession number MSV000101115 (https://doi.org/10.25345/C5QB9VK4S). All the other data are available within the article and its supplementary information.
