# Supplementary material for: Extracellular wax ester biosynthesis by staphylococcal lipases detoxifies skin fatty acids and shapes interspecies competition
Source: ISME J. 2026 Jun 11;20(1):wrag148. doi: 10.1093/ismejo/wrag148 (PMC13521525; doi:10.1093/ismejo/wrag148)
Supplement: Supplementary file 1 [file we_paper_supplementary_files_r2_wrag148.docx]

**Supplementary information for**

**Extracellular wax ester biosynthesis by staphylococcal lipases detoxifies skin fatty acids and shapes interspecies competition**

Justine Camus^1,2,3^, Christian D. Freeman^4^, Xuanheng Hu^1,2,3^, Aaron Komnik^1,2,3^, Rebekah L. Casey^4^, David T. Brewer^4^, Soobin Yang^1,2,3^, Kelly M. Hines^4^, Arnaud Kengmo Tchoupa^1,2,3,^*

^1^ Interfaculty Institute of Microbiology and Infection Medicine Tübingen, Chair of Infection Biology, University of Tübingen, Tübingen, Germany

^2^ Cluster of Excellence EXC 2124 Controlling Microbes to Fight Infections, University of Tübingen, Tübingen, Germany

^3^ German Centre for Infection Research (DZIF), partner site Tübingen

^4^ Department of Chemistry, University of Georgia, Athens, GA, USA

* Corresponding author: Arnaud Kengmo Tchoupa. IMIT – Infection Biology, University of Tübingen, Auf der Morgenstelle 28, 72076, Tübingen, Germany. E-mail: ([arnaud.kengmo-tchoupa@uni-tuebingen.de](mailto:arnaud.kengmo-tchoupa@uni-tuebingen.de))

This file includes:

- Supplementary text
- Tables S1 to S3
- Fig. S1 to S16

**Supplementary text**

**Growth media and conditions**

Unless otherwise specified, *S. aureus, S. epidermidis, S. simulans* and *Escherichia coli* strains were routinely cultured under aerobic conditions overnight at 37 °C with shaking (160 rpm) in tryptic soy broth (TSB, Oxoid) or lysogeny broth (LB, Sigma). Whenever appropriate, the medium was supplemented with ampicillin (100 μg/mL), kanamycin (30 μg/mL), or chloramphenicol (10 μg/mL).

**Growth and spot assays**

To assess the effects of antimicrobial fatty acids (AFAs), long-chain fatty alcohols (FOH), and wax esters (WE) on *S. aureus* replication, overnight cultures were diluted (1:1000) in basic medium [1] (BM; 1% soy peptone, 0.5% yeast extract, 0.5% NaCl, 0.1% glucose, 0.1% K_2_HPO_4_, pH 7.2), BM supplemented with 1.5% NaCl, chemically defined medium (CDM [2]), or nutrient broth no.2 (NB, Oxoid). Bacteria were grown in 96-well plates (U-bottom) for 24 h at 37°C with linear shaking at 567 cpm (3-mm excursion) in plain medium or medium supplemented with lipids. Optical density at 600 nm (OD_600_) was recorded every 15 min using an Epoch 2 microplate reader (BioTek). Growth curves and area under growth curves were generated using GraphPad Prism version 10.6.1 [3]. Upon 24 h incubation, cultures were serially diluted, and 5 µL of each dilution were spotted onto tryptic soy agar (TSA). Plates were incubated overnight at 37 °C and subsequently imaged.

**Total RNA purification and quantitative real-time PCR analysis of gene expression**

Overnight cultures of *S. aureus* USA300 JE2 in basic medium (BM) were diluted to an OD_600_ of 0.1 in 50 mL BM. Bacteria were left untreated or treated with 20 µM palmitoleic acid (PA), 20 µM palmitoleyl alcohol (POH), or a combination of 20 µM PA and 20 µM POH during incubation at 37°C until an OD_600_ of ~2.5 was reached. This was achieved after approximately 2 h for bacteria grown in plain BM, whereas lipid-supplemented cultures required roughly 3.5 h. Next, bacteria were harvested by centrifugation (15 min, 4,000 × *g*, 4°C). Pellets were resuspended in 1 mL TRI Reagent (Sigma), transferred to tubes containing 0.5 mL zirconium silica beads (Roth), and cells were disrupted using a high-speed homogenizer (Precellys Evolution Touch, Bertin Technologies). Chloroform (200 µL) was added to the lysates prior to centrifugation (15 min, 12,000 × *g*, 4°C). The aqueous phase was mixed with isopropanol and incubated for 10 min at room temperature before RNA precipitation by centrifugation (30 min, 12,000 × *g*, 4°C). RNA pellets were washed with 70% ethanol and resuspended in RNase-free water. Residual genomic DNA was removed by DNase treatment (Thermo Scientific) for 3 h, followed by RNA purification using the NucleoSpin RNA Clean-up kit (Macherey-Nagel).

Primers used for quantitative reverse transcription PCR (qRT-PCR) are listed in Table S3. qRT-PCR was performed using the One-Step TB Green PrimeScript RT-PCR Kit (TaKaRa) according to the manufacturer’s instructions. Reactions were carried out on a QuantStudio 3 system (Thermo Scientific) using three independent biological replicates. Expression levels were normalized to the housekeeping gene *gyrB*.

**Lipase activity assay**

Lipolytic activity of *S. aureus* and coagulase-negative staphylococci was measured using fluorogenic 4-methylumbelliferyl ester substrates (Biosynth) as described elsewhere [4]. Briefly, bacteria-conditioned media were diluted fifty times with the assay buffer (PBS, 0.01% Triton X-100) supplemented with 100 µM substrate. After incubation for 30 min at 37 °C in the dark, fluorescence (excitation wavelength = 365 nm, emission wavelength = 455 nm) resulting from lipase-mediated release of 4-methylumbelliferone was measured using a CLARIOStar microplate reader (BMG LabTech).

**Biofilm formation assay**

Overnight bacterial cultures were diluted to an OD_600_ of 0.03 (or an OD_600_ of 3, where indicated) in plain medium or medium supplemented with lipids before incubation for 6 or 24 h at 37°C without agitation in sealed 24-well cell culture plates (Greiner). After incubation, non-adherent cells were removed, and biofilms were washed with PBS. Next, biofilms were dried for 30 min and then stained with 0.1% safranin in 50% ethanol, as previously described [1,5]. After 15 min, biofilms were washed with PBS, photographed, and safranin was eluted for 15 min with a solution of 70% ethanol and 10% isopropanol. Biofilm biomass was quantified by measuring absorbance at 530 nm (OD_530_) using a CLARIOStar microplate reader.

**Purification of recombinant, staphylococcal lipases**

Staphylococcal lipases Lip1, Lip2, SsL, GehC, and GehD were purified as previously described [6]. DNA sequences encoding the mature form of each lipase were amplified and cloned in frame with the N-terminal His_6_ tag in pET28a(+). Proteins were overexpressed in *Escherichia coli* BL21 (DE3) grown in LB and induced with 0.5 mM IPTG once cultures reached an OD_600_ of 0.6 - 0.8 at 25 °C. Bacterial cells were harvested by centrifugation (11,800 × *g*, 4°C) and resuspended in lysis buffer (50 mM Tris-HCl, 300 mM NaCl, 10 mM imidazole, pH 8.0) supplemented with EDTA-free protease inhibitor cocktail (Roche) and DNase (Sigma), and kept on ice. Cells were lysed by two cycles of sonication (3 min; 0.5 s on/0.5 s off; 40% amplitude). After centrifugation (17,000 rpm for 45 min at 4 °C), supernatants were filtered through 0.2 µm filters (VWR) and applied to HisTrap FF™ crude columns (Cytiva) for affinity purification. After washing with buffer containing 2 to 20 % elution buffer (50 mM Tris-HCl, 300 mM NaCl, 500 mM imidazole), proteins were eluted with 50 to 100 % elution buffer. Eluted proteins were dialyzed overnight at 4 °C with 10 mM Tris-HCl, 200 mM NaCl, and subsequently subjected to size exclusion chromatography using a HiLoad 26/60 Superdex 200 pg column on an ÄKTAgo system (Cytiva). Purified proteins were stored at - 80 °C.

**Lipid extraction and high-performance thin layer chromatography**

Recombinant lipases (1 µg/mL) of *S. aureus* (Lip1, Lip2, or the catalytic mutant Lip2^S412A^) *S. simulans* (SsL) and *S. epidermidis* (GehC or GehD), were incubated in 300 µL of 0.1 M sodium phosphate buffer (pH 6.0) supplemented with equimolar concentrations (300 µM) of AFAs and FOH, or 600 µM of the wax ester palmitoleyl palmitoleate in glass vials and incubated for 18 to 24 h at 37°C with shaking (160 rpm). Reactions were terminated by addition of chloroform:methanol (2:1, v/v), and lipids were extracted as described elsewhere [1,7]. After 30 min of incubation with shaking and subsequent centrifugation, the organic phase was collected, transferred to clean glass vial, dried, and resuspended in chloroform:methanol (2:1, v/v). Lipid extracts were applied to silica gel high-performance thin-layer chromatography (HPTLC) plates (silica gel 60 F_254_, Merck) using a Linomat 5 sample applicator (CAMAG). Lipids were separated in an automated developing chamber (ADC 2, CAMAG) using petroleum ether:ethyl ether:acetic acid (90:10:1, v/v/v) as the mobile phase. Lipid species were visualized in an iodine vapor chamber. Palmitoleic acid (PA), palmitoleyl alcohol (POH), and palmitoleyl palmitoleate (WE) were used as lipid standards.

**Cocultivation assays**

Empty-vector (EV)-bearing *S. aureus* USA300 JE2 WT or its isogenic Δ*lip1/2* mutant was grown either alone or in coculture at a 1:1 ratio, as determined by OD_600_ measurement, with *S. simulans* ATCC 27848 (WT or its isogenic lipase-deficient Δ*ssL* mutant), or *S. epidermidis* 1457. Bacteria were cultured with shaking (planktonic growth) or without shaking to allow biofilm formation. For planktonic growth, monocultures or cocultures were performed in basic medium (BM) or BM supplemented with 200 µM palmitic acid (PA), or in BM supplemented with 200 µM PA and 200 µM palmitoleyl alcohol (POH). Samples in 96-well plates were then incubated at 37°C with shaking for 24 h in an Epoch 2 microplate reader. In contrast, biofilms were formed in a static incubator (24 h at 37°C) in plain medium (BM, BM supplemented with 1.5% NaCl or CDM) or in medium supplemented with 50 µM (CDM) or 100 µM (BM or BM + 1.5% NaCl) PA, or in medium supplemented with equimolar concentrations (50 or 100 µM) of PA and POH. After washing with PBS to remove non-adherent bacteria, biofilms were detached and resuspended in PBS via multiple pipetting up and down. Planktonic and biofilm monocultures and cocultures were serially diluted. Next, using a spiral plater (Eddy Jet 2, IUL), diluted monocultures were plated on tryptic soy agar (TSA), whereas diluted cocultures were plated on both TSA and TSA supplemented with 10 µg/mL chloramphenicol. Following overnight incubation, colony-forming units (CFU) were quantified using an automated colony counter (Flash & Go, software version 1.1.1104.0, IUL). The limit of detection was 203 CFU/mL. The CFU count on a TSA plate reflected total number of viable bacteria in a sample (*S. aureus* and/or CoNS), whereas the CFU count on TSA + chloramphenicol corresponded specifically to empty vector-bearing and thereby chloramphenicol-resistant *S. aureus*. The relative abundance of each bacterial species in coculture was calculated using these CFU counts. A coculture assay as described above was also performed with plasmid-free *S. aureus* USA300 JE2 strains (WT vs Δ*lip1/2*), except that TSA + erythromycin plates were used to discriminate between erythromycin-resistant Δ*lip1/2* and its WT.

**Biofilm invasion assay**

Overnight cultures of wild-type *S. simulans* or its lipase-deficient mutant (Δ*ssL*) were diluted to an OD_600_ of 0.03 in basic medium (BM) supplemented with 1.5% NaCl and incubated for 24 h at 37°C to allow biofilm formation. Biofilms were subsequently washed with PBS to remove non-adherent bacteria prior to the addition of fresh medium (BM + 1.5% NaCl) or medium supplemented with 100 µM PA, or 100 µM PA + 100 µM POH. For cocultivation experiments, overnight cultures of empty-vector (EV)-bearing *S. aureus* USA300 JE2 WT or Δ*lip1/2* strains were diluted to an OD_600_ of 0.03 and added to preformed *S. simulans* biofilms. Following an additional 24 h incubation at 37°C, biofilms were washed, resuspended, serially diluted, and plated on tryptic soy agar (TSA) for monocultures and cocultures, and on TSA supplemented with 10 µg/mL chloramphenicol for cocultures only, as described for the cocultivation assays, allowing CFU enumeration and the calculation of relative abundance.

**Table S1. Bacterial strains, plasmids and phage.**

| **Strain** | **Description** | **Reference** |
| --- | --- | --- |
| ***Staphylococcus aureus*** | | |
| Newman | Methicillin-sensitive *S. aureus* (MSSA) | [8] |
| SH1000 | NCTC8325 derivative with a functional *rsbU* gene, Δ*tcaR*, cured of Φ11, Φ12, and Φ13, MSSA | [9] |
| USA200 UAMS-1 | MSSA | [10] |
| USA300 JE2 | Community-acquired methicillin-resistant *S. aureus* (CA-MRSA), plasmid-cured derivative of the USA300 LAC strain | [11] |
| USA300 JE2 Δ*lip1/2* | USA300 JE2 defective for Lip1 and Lip2 | [12] |
| USA400 MW2 | CA-MRSA | [13] |
| ***Staphylococcus simulans*** | | |
| ATCC27848 | Human skin isolate | [14] |
| ATCC27848 Δ*ssL* | ATCC27848 defective for SsL | This study |
| ***Staphylococcus epidermidis*** | | |
| 1457 | Clinical isolate from central venous catheter infection, *ica-* and *aap*-positive | [15] |
| ***Escherichia coli*** | | |
| IM08B | SA08BΩP_N25_-*hsdS* (CC1-1) of MW2 integrated between the *essQ* and *cspB* genes | [16] |
| BL21 (DE3) | F^-^ *ompT hsdS_B_*(r_B_^-^m_B_^-^) *gal dcm* (DE3), IPTG-inducible T7 RNA polymerase | [17] |
| **Plasmids** | | |
| pALC2073 | *E. coli-S. aureus* shuttle vector : contains P_xyl/tet_ ; Amp^R^ and Cm^R^ | [18] |
| p*lip1* | pALC2073 containing the *S. aureus* *lip1* coding region | [1] |
| p*lip2* | pALC2073 containing the *S. aureus* *lip2* coding region | [19] |
| p*ssL* | pALC2073 containing the *S. simulans* *ssL* coding region | This study |
| p*gehC* | pALC2073 containing the *S. epidermidis* *gehC* coding region | This study |
| p*gehD* | pALC2073 containing the *S. epidermidis* *gehD* coding region | This study |
| pIMAY | Temperature-sensitive allelic exchange plasmid for staphylococci | [20] |
| pIMAY-*ssL* | pIMAY carrying the *ssL* deletion construct | This study |
| pET28a(+)-*lip1* | pET28a(+) for *S. aureus* Lip1 expression | This study |
| pET28a(+)-*lip2* | pET28a(+) for *S. aureus* Lip2 expression | This study |
| pET28a(+)-*ssL* | pET28a(+) for *S. simulans* SsL expression | This study |
| pET28a(+)-*gehC* | pET28a(+) for *S. epidermidis* GehC expression | This study |
| pET28a(+)-*gehD* | pET28a(+) for *S. epidermidis* GehD expression | This study |
| **Phage** | | |
| Φ187 | Siphovirus used for plasmid transfer between *S. aureus* and CoNS | [21] |

**Supplemental references**

1.	Kengmo Tchoupa A, Elsherbini AMA, Camus J, et al. Lipase-mediated detoxification of host-derived antimicrobial fatty acids by Staphylococcus aureus. *Commun Biol*. May 15 2024;7(1):572. doi:10.1038/s42003-024-06278-3

2.	Pohl K, Francois P, Stenz L, et al. CodY in Staphylococcus aureus: a regulatory link between metabolism and virulence gene expression. *J Bacteriol*. May 2009;191(9):2953-63. doi:10.1128/JB.01492-08

3.	Kengmo Tchoupa A, Peschel A. Staphylococcus aureus Releases Proinflammatory Membrane Vesicles To Resist Antimicrobial Fatty Acids. *mSphere*. Sep 30 2020;5(5)doi:10.1128/mSphere.00804-20

4.	Lentz CS, Sheldon JR, Crawford LA, et al. Identification of a S. aureus virulence factor by activity-based protein profiling (ABPP). *Nat Chem Biol*. Jun 2018;14(6):609-617. doi:10.1038/s41589-018-0060-1

5.	Cheung GY, Yeh AJ, Kretschmer D, et al. Functional characteristics of the Staphylococcus aureus delta-toxin allelic variant G10S. *Sci Rep*. Dec 10 2015;5:18023. doi:10.1038/srep18023

6.	Joiner JD, Steinchen W, Mozer N, et al. HilE represses the activity of the Salmonella virulence regulator HilD via a mechanism distinct from that of intestinal long-chain fatty acids. *J Biol Chem*. Dec 2023;299(12):105387. doi:10.1016/j.jbc.2023.105387

7.	Bligh EG, Dyer WJ. A rapid method of total lipid extraction and purification. *Can J Biochem Physiol*. Aug 1959;37(8):911-7. doi:10.1139/o59-099

8.	Duthie ES, Lorenz LL. Staphylococcal coagulase; mode of action and antigenicity. *J Gen Microbiol*. Feb 1952;6(1-2):95-107. doi:10.1099/00221287-6-1-2-95

9.	Horsburgh MJ, Aish JL, White IJ, Shaw L, Lithgow JK, Foster SJ. sigmaB modulates virulence determinant expression and stress resistance: characterization of a functional rsbU strain derived from Staphylococcus aureus 8325-4. *J Bacteriol*. Oct 2002;184(19):5457-67. doi:10.1128/jb.184.19.5457-5467.2002

10.	Sassi M, Sharma D, Brinsmade SR, Felden B, Augagneur Y. Genome Sequence of the Clinical Isolate Staphylococcus aureus subsp. aureus Strain UAMS-1. *Genome Announc*. Feb 12 2015;3(1)doi:10.1128/genomeA.01584-14

11.	Fey PD, Endres JL, Yajjala VK, et al. A genetic resource for rapid and comprehensive phenotype screening of nonessential Staphylococcus aureus genes. *mBio*. Feb 12 2013;4(1):e00537-12. doi:10.1128/mBio.00537-12

12.	Nguyen MT, Luqman A, Bitschar K, et al. Staphylococcal (phospho)lipases promote biofilm formation and host cell invasion. *Int J Med Microbiol*. Aug 2018;308(6):653-663. doi:10.1016/j.ijmm.2017.11.013

13.	Wang R, Braughton KR, Kretschmer D, et al. Identification of novel cytolytic peptides as key virulence determinants for community-associated MRSA. *Nat Med*. Dec 2007;13(12):1510-4. doi:10.1038/nm1656

14.	Kloos WE, Schleifer KH. Isolation and Characterization of Staphylococci from Human Skin II. Descriptions of Four New Species: Staphylococcus warneri, Staphylococcus capitis, Staphylococcus hominis, and Staphylococcus simulans. *International Journal of Systematic and Evolutionary Microbiology*. 1975;25(1):62-79. doi:10.1099/00207713-25-1-62

15.	Mack D, Siemssen N, Laufs R. Parallel induction by glucose of adherence and a polysaccharide antigen specific for plastic-adherent Staphylococcus epidermidis: evidence for functional relation to intercellular adhesion. *Infect Immun*. May 1992;60(5):2048-57. doi:10.1128/iai.60.5.2048-2057.1992

16.	Monk IR, Tree JJ, Howden BP, Stinear TP, Foster TJ. Complete Bypass of Restriction Systems for Major Staphylococcus aureus Lineages. *mBio*. May 26 2015;6(3):e00308-15. doi:10.1128/mBio.00308-15

17.	Studier FW, Moffatt BA. Use of bacteriophage T7 RNA polymerase to direct selective high-level expression of cloned genes. *J Mol Biol*. May 5 1986;189(1):113-30. doi:10.1016/0022-2836(86)90385-2

18.	Bateman BT, Donegan NP, Jarry TM, Palma M, Cheung AL. Evaluation of a tetracycline-inducible promoter in Staphylococcus aureus in vitro and in vivo and its application in demonstrating the role of sigB in microcolony formation. *Infect Immun*. Dec 2001;69(12):7851-7. doi:10.1128/IAI.69.12.7851-7857.2001

19.	Cadieux B, Vijayakumaran V, Bernards MA, McGavin MJ, Heinrichs DE. Role of lipase from community-associated methicillin-resistant Staphylococcus aureus strain USA300 in hydrolyzing triglycerides into growth-inhibitory free fatty acids. *J Bacteriol*. Dec 2014;196(23):4044-56. doi:10.1128/JB.02044-14

20.	Monk IR, Shah IM, Xu M, Tan MW, Foster TJ. Transforming the untransformable: application of direct transformation to manipulate genetically Staphylococcus aureus and Staphylococcus epidermidis. *mBio*. 2012;3(2)doi:10.1128/mBio.00277-11

21.	Winstel V, Kuhner P, Rohde H, Peschel A. Genetic engineering of untransformable coagulase-negative staphylococcal pathogens. *Nat Protoc*. May 2016;11(5):949-59. doi:10.1038/nprot.2016.058

**Table S2. Primers used for cloning and genetic manipulation**

| **Oligonucleotide** | **Sequence (5’ – 3’)** |
| --- | --- |
|  | |
| For cloning p*ssL* (pALC2073-*ssL*): | |
| KpnI-*ssL*-fwd | GCTAGGTACCGAATAGGAGCAATTCAAAATG |
| SacI-*ssL*-rev | GCGCGAGCTCTTAGATATCTAATTCTTCTAT |
|  |  |
| For cloning p*gehC* (pALC2073-*gehC*): | |
| KpnI-*gehC*-fwd | GCTAGGTACCTAGATAGAGGTGCTAATTGTG |
| SacI-*gehC*-rev | GCGCGAGCTCTTATTTATTTGTTGATGTTAA |
|  |  |
| For cloning p*gehD* (pALC2073-*gehD*): | |
| KpnI-*gehD*-fwd | GCTAGGTACCCTTATAGAGGTGATTTTTTTG |
| SacI-*gehD*-rev | GCTAGAGCTCCTACTTACGTGTAATACC |
|  |  |
| For cloning pET28a(+)-*lip1* (*lip1* is also known as *lip*): | |
| NheI-mp *lip1*-fwd | GAACGCTAGCAAAGTTGCCAAACAAGGG |
| SacI-*Sal1*-rev | GGTAGAGCTCTTATGCTTGCTTAGTATCAG |
|  |  |
| For cloning pET28a(+)-*lip2* (*lip2* is also known as *geh*): | |
| NheI-mp *lip2*-fwd | GGACGCTAGCCTTAAAGCGAATCAAGTAC |
| SacI-*gehB*-rev | CCAGGAGCTCTTAACTTGCTTTCAATTGTGTTCC |
|  |  |
| For cloning pET28a(+)-*ssL* (*ssL* is also known as *lip*): | |
| NheI-mp *lip sim*-fwd | GCGCGCTAGCAAAACTAATAAAGCGCAAC |
| SacI-*ssL*-rev | GCGCGAGCTCTTAGATATCTAATTCTTCTAT |
|  |  |
| For cloning of pET28a(+)-*gehC*: | |
| NheI-mp *gehC*-fwd | GCGCGCTAGCAAATCAGCAAAACAAAAAC |
| SacI-*gehC*-rev | GCGCGAGCTCTTATTTATTTGTTGATGTTAA |
|  |  |
| For cloning of pET28a(+)-*gehD*: | |
| NheI-mp *gehD*-fwd | GAACGCTAGCCAACTTACTGCGCAAGCTC |
| SacI-*gehD*-rev | GCTAGAGCTCCTACTTACGTGTAATACC |
|  |  |
| For deletion of *ssL* (pIMAY-Δ*ssL*) | |
| KpnI-*ssL* ups-fwd | CAGTCAGGTACCAATTGATGAAACACGACC |
| SOEING *ssL* ups-rev | TATTGATTAGATATCTAATTCATTATTTTTCTTCATTTTG |
| *ssL* dwns-fwd | GAATTAGATATCTAATCAATA |
| SacI-*ssL* dwns-rev | CAGTCAGAGCTCCACGAAACTCTGGCTATG |

**Table S3.** **Primers used for quantitative reverse transcription PCR.**

| Gene | Oligonucleotide sequence (5’ – 3’) |
| --- | --- |
| *gyrB* | Forward: GGTGGCGACTTTGATCTAGC |
|  | Reverse: TTATACAACGGTGGCTGTGC |
|  |  |
| *lip2* | Forward: CACCAACTTCAACTACACCC |
|  | Reverse: TACGTTTCGTTCTACTATGAC |
|  |  |
| *psmα* | Forward CATCGTTTTGTCCTCCTG |
|  | Reverse: TCATCGCTGGCATCATTA |
|  |  |
| *hla* | Forward: AGAAAATGGCATGCACAAAAA |
|  | Reverse: TATCAGTTGGGCTCTCTAAAA |
|  |  |
| *farE* | Forward: CATGGATAGTGATTGTAGCTG |
|  | Reverse: CAATACCATCATCTTTAGTCGTC |
|  |  |
| *saeR* | Forward: CCATTTACGCCTTAACTTTA |
|  | Reverse: TAGTCATATCCCCAAACTT |
|  |  |
| *isdA* | Forward: GCAGTTACAGCAGGTTTA |
|  | Reverse: CAGCAAAACCAAACAATG |
|  |  |
| *clfA* | Forward: CGGTTTTGGACTACTCAGCA |
|  | Reverse: TAGCTTCACCAGTTACCGGC |
|  |  |
| *clfB* | Forward: ATAGGCAATCATCAAGCA |
|  | Reverse: TGTATCATTAGCCGTTGTAT |
|  |  |
| *fnbA* | Forward: GGAGCAGCATCAGTATTCTT |
|  | Reverse: AGTTGCAGTTGTTTGTGTTT |

**Figure S1. Fatty alcohols and fatty acids induce transcriptional changes in *S. aureus*.**

*S. aureus* USA300 JE2 was grown in basic medium (BM) or in BM supplemented with 20 µM palmitoleic acid (PA), 20 µM palmitoleyl alcohol (POH), or both 20 µM PA and 20 µM POH until an optical density (OD_600_) of ~2.5 was reached. Following total RNA extraction from three experimentally independent biological replicates, quantitative reverse transcription PCR was performed to analyse the expression of *lip2, psmα, clfA, fnbA, hla* (**A**), *saeR, isdA, clfB*, and *farE* (**B**). Values were normalized to *gyrB* expression. Means ± SD (error bars) are plotted. *, *P* < 0.05; **, *P* < 0.01; ***, *P* < 0.001; ****, *P* < 0.0001 per two-way ANOVA with Dunnett’s multiple comparisons test relative to the control sample (-).

**Figure S2. Fatty alcohols protect *S. aureus* from AFAs in a lipase-dependent manner.**

Area under the growth curves of wild-type USA300 JE2 (**A, B, C**, **D**, and **E**) and its isogenic lipase-deficient

mutant Δ*lip1/2* (**A**, **B,** **D**, and **E**) grown for 24 h in plain medium [nutrient broth (NB) for panel **A** or basic medium (BM) for panels **B, C**, **D**, and **E**], medium supplemented with fatty acids [palmitoleic acid (PA), sapienic acid (SA), linoleic acid (LA), or palmitic acid (Pa)], medium supplemented with fatty alcohols [palmitoleyl alcohol (POH), linoleyl alcohol (LOH), α-linolenyl alcohol (ALOH) or arachidonyl alcohol (AOH)], or medium supplemented with indicated fatty acids and fatty alcohols. Unless otherwise specified, bacteria were treated with 50 µM of fatty acids and/or 50 µM of fatty alcohols. Data shown are means ± SD for three or four biological replicates. Statistical significance was determined by two-way ANOVA with Tukey’s multiple comparisons test (**A-B**) or with Dunnett’s multiple comparisons test relative to PA-treated sample (**D**, and **E**). ****, *P* < 0.0001.

**Figure S3. Wild-type *S. aureus* provides fatty alcohol-dependent cross-protection to its lipase-deficient mutant against AFAs.**

Wild-type (WT) *S. aureus* USA300 JE2 (**A**) or its isogenic Δ*lip1/2* mutant (**B**) was cultured alone or co-cultured together in basic medium (BM), BM with palmitoleic acid (PA – 50 µM), or BM with PA and palmitoleyl alcohol (POH – 50 µM). After 24 h incubation, cultures were serially diluted and selectively plated for colony-forming unit (CFU) counting. CFU counts (**A** or **B**) were used to calculate the relative abundance (**C**) of *S. aureus* (WT or Δ*lip1/2*). Data shown are means ± SD of three biological replicates. Statistical significance was determined by two-way ANOVA with Tukey’s multiple comparisons test (**A-B**). ****, *P* < 0.0001. LOD indicates the limit of detection [log10(203 CFU/mL) = 2.3].

**Figure S4. Lipolytic activity of staphylococcal lipases.**

**A.** Amino acid sequence alignment of the mature forms of staphylococcal lipases (Lip1 and Lip2 from *S. aureus*, SsL from *S. simulans*, and GehC and GehD from *S. epidermidis*) generated using Clustal Omega.

**B.** Conditioned media from cultures of empty-vector (EV)-bearing Δ*lip1/2* mutant or Δ*lip1/2* complemented with p*lip1*, p*lip2*, p*ssL*, p*gehC* or p*gehD* were incubated for 30 min with fluorogenic substrates. The release of 4-methylumbelliferone, indicative of lipolytic activity, was quantified by measuring fluorescence (λ = 455 nm). Substrates used are 4-methylumbelliferyl acetate (2:0), 4-methylumbelliferyl butyrate (4:0), 4-methylumbelliferyl caprylate (8:0), and 4-methylumbelliferyl oleate (18:1). Data shown are means ± SD of three biological replicates. Statistical significance was determined by two-way ANOVA with Dunnett’s multiple comparisons test relative to EV-carrying Δ*lip1/2* mutant. ****, *P* < 0.0001.

**Figure S5. AFA-mediated inhibition of biofilm formation by *S. aureus* is alleviated by fatty alcohol in a lipase-dependent manner.**

Wild-type USA300 JE2 and its isogenic Δ*lip1/2* mutant carrying empty vector (EV) or Δ*lip1/2* complemented with p*lip1* or p*lip2* (**A, C**), or EV-bearing Δ*lip1/2* and Δ*lip1/2* complemented with p*ssL*, p*gehC* or p*gehD* (**B**) were grown for 24 h in basic medium (BM), BM supplemented with palmitoleic acid (PA) or BM with equimolar concentrations of PA and palmitoleyl alcohol (POH – 100 µM for panels **A** and **B**, 75 µM for panel **C**) to form biofilms. Biofilms were stained with 0.1% safranin, photographed (**A-B**) and/or optical density at 530 nm (OD_530_) was measured (**C**). **A-B.** One representative biological replicate (n = 3) is shown. **C.** Data are presented as means ± SD of three biological replicates. Statistical significance was determined by two-way ANOVA with Tukey’s multiple comparisons test. **, *P* < 0.01; ****, *P* < 0.0001.

**Figure S6. Fatty alcohols promote *S. aureus* attachment during biofilm formation.**

**A.** Wild-type (WT) USA300 JE2 and its isogenic Δ*lip1/2* mutant carrying an empty vector (EV) or Δ*lip1/2* complemented with p*lip1*, p*lip2*, p*ssL*, p*gehC*, or p*gehD*, were grown for 24 h in basic medium (BM) or in BM supplemented with 100 µM palmitoleyl alcohol (POH) to form biofilms. **B-C.** Overnight cultures of WT JE2 and Δ*lip1/2* mutant were diluted to an optical density (OD_600_) of 0.03 (**B**) or an OD_600_ of 3 (**C**) in BM or BM with 100 µM POH prior to a 6 h incubation under static conditions to allow biofilm formation. Biofilms were stained with 0.1% safranin and OD_530_ was measured to quantify biofilm formation. Data are presented as means ± SD of three biological replicates. Statistical significance was determined by two-way ANOVA with Šídák’s (**A**) or with uncorrected Fisher’s LSD (**B-C**) multiple comparisons test. *, *P* < 0.05.

**Figure S7. Phosphatidylglycerol levels of lipase-expressing mutants mirror their wax ester production abilities.**

Empty vector (EV)-carrying USA300 JE2 Δ*lip1/2* mutant or Δ*lip1/2* complemented with p*lip1*, p*lip2*, p*ssL*, p*gehC* or *pgehD* were grown for 24 h with 300 µM palmitoleic acid and 300 µM palmitoleyl alcohol. After 24 h incubation, bacterial pellets were processed for lipidomic analysis using hydrophilic interaction liquid chromatography ion-mobility mass spectrometry in negative ion mode [M-H]^-^. Phosphatidylglycerols (PG) were measured. **A.** PG 31:1 (*m/z* 705.487). **B.** PG 32:2 (*m/z* 717.472). **C.** PG 33:1 (*m/z* 733.503). **D.** PG 34:2 (*m/z* 745.503). **E.** PG 35:1 (*m/z* 761.523). **F.** PG 36:1 (*m/z* 775.547). **G.** PG 31:0 (*m/z* 707.485). **H.** PG 32:0 (*m/z* 721.503). **I.** PG 34:0 (*m/z* 749.534). Data shown are means ± SD (*n* = 3).

**Figure S8. Recombinant lipases esterify fatty alcohols with AFAs to generate WE.**

Representative (n = 3) extracted ion chromatogram (XIC) of wax ester (WE) palmitoleyl palmitoleate 32:2 (*m/z* 477.466) (**A**) or palmitoleic acid 16:1 (PA – *m/z* 253.217) (**B**) upon 24 h incubation of 100 µM PA with 100 µM palmitoleyl alcohol (POH) and 1 µg/mL of a recombinant staphylococcal lipase (Lip1, Lip2, SsL, GehC or GehD, as indicated). After lipid extraction, WE and PA were measured via hydrophilic interaction liquid chromatography ion-mobility mass spectrometry in positive ([M+H]^+^ – **A**) or negative ([M-H]^-^ – **B**) ion modes.

**C.** Recombinant lipases (1 ng/µL) as detailed in (**A-B**) were incubated for 24 h with 300 µM PA and 300 µM POH. High-performance thin-layer chromatography (HPTLC) was performed to separate lipid extracts.

**D-H.** Recombinant (1 ng/µL) Lip2 (**D-H**), Lip1 (**E, F**), or catalytically inactive Lip2 (Lip2^S412A^ – **G and H**) were incubated with 300 µM antimicrobial fatty acids (AFAs) such as PA (**D, G**), oleic acid (**E, G**), sapienic acid (**G**), linoleic acid (**E, H**), α-linolenic acid (**F, H**) and arachidonic acid (**F, H**) for 24 h. Wherever indicated, samples were supplemented with 300 µM fatty alcohols like POH (**D, E, F, G and H**) , linoleyl alcohol (LOH - **D**), α-linolenyl alcohol (ALOH - **D**) or arachidonyl alcohol (AOH - **D**). HTPLC was used to separate lipids.

**Figure S9. Wax esters do not interfere with bacterial growth, AFA toxicity or Lip2-mediated protection of fatty alcohol.**

**A-C.** High-performance thin-layer chromatography of lipids extracted after 24 h incubation of 600 µM wax ester (WE) palmitoleyl palmitoleate with recombinant lipases Lip1, Lip2, SsL, GehC and GehD (1 ng/µL) in phosphate buffer pH 6 (**A**), pH 7 (**B**) or pH 8 (**C**). WE hydrolysis led to the detection of bands corresponding to palmitoleic acid (PA) and palmitoleyl alcohol (POH).

**D-E.** EV-bearing wild-type USA300 JE2 and Δ*lip1/2* or Lip1- or Lip2-proficient Δ*lip1/2* mutant (complemented with p*lip1* or p*lip2*, respectively) were cultivated in chemically defined medium (CDM), CDM supplemented with PA, WE, PA and POH, PA and WE, or PA + POH + WE (50 µM of each lipid unless otherwise specified) for 24 h. Area under the growth curves are shown in arbitrary units (AU). Data shown are means ± SD of three biological replicates. Statistical significance was determined by two-way ANOVA with Dunnett’s multiple comparisons test relative to PA-treated sample. ****, *P* < 0.0001.

**Figure S10. Fatty alcohols protect lipase-proficient *S. simulans* from AFA toxicity.**

**A-B.** Conditioned media from wild-type *S. simulans* or its isogenic lipase-deficient Δ*ssL* mutant (**A**) or from *S. epidermidis* (**B**) were incubated for 30 min with fluorogenic substrates. Fluorescence (λ = 455 nm) was measured to quantify lipase activity. Substrates used are 4-methylumbelliferyl acetate (2:0), 4-methylumbelliferyl butyrate (4:0), 4-methylumbelliferyl caprylate (8:0) and 4-methylumbelliferyl oleate (18:1).

**C-D*.*** Wild-type (WT) *S. simulans* (**C**) or *S. epidermidis* (**D**) was cultured alone or co-cultured with *S. aureus* (WT or lipase-deficient mutant Δ*lip1/2*) in basic medium (BM), BM with 200 µM palmitoleic acid (PA), or BM with 200 µM PA and 200 µM palmitoleyl alcohol (POH). After 24 h incubation, cultures were selectively plated for colony-forming unit (CFU) counting. LOD indicates the limit of detection [log10(203 CFU/mL) = 2.3]. Data shown are means ± SD of three or four biological replicates. Two-way ANOVA (**A**, **C**, and **D**), with Šídák’s (**A**) or Tukey’s multiple comparisons test (**C** and **D**), and one-way ANOVA with Tukey’s multiple comparisons test (**B**) were used to determined statistical significance. *, *P* < 0.05; **, *P* < 0.01; ***, *P* < 0.001; ****, *P* < 0.0001.

**Figure S11.** **FOH protect *S. simulans* from AFAs without SsL.**

**A-E.** Wild-type (WT) *S. aureus* USA300 JE2 or its Δ*lip1/2* mutant was cultured alone or co-cultured with WT *S. simulans* or its SsL-deficient mutant *(S. simulans* Δ*ssL*) in basic medium (BM), BM with 200 µM palmitoleic acid (PA), or BM with 200 µM PA and 200 µM palmitoleyl alcohol (POH) for 24 h. Cultures were then serially diluted for selective plating and colony-forming unit (CFU) enumeration. CFU counts (**A, D** and **E**) were used to calculate the relative abundance *S. aureus* Δ*lip1/2* and WT *S. simulans* (**B**), or Δ*lip1/2* and *S. simulans* Δ*ssL* (**C**). **F-G.** WT *S. simulans*, *S. simulans* Δ*ssL*, or *S. simulans* Δ*ssL* supplemented with recombinant SsL (1 ng/µL) were cultivated alone or co-cultivated with *S. aureus* Δ*lip1/2* mutant as described in **A-E**. Plotted CFU counts of WT *S. simulans* (**F**) and its isogenic Δ*ssL* mutant are presented. Data shown are means ± SD of three biological replicates. Statistical significance was determined by two-way ANOVA with Tukey’s multiple comparisons test. **, *P* < 0.01; ***, *P* < 0.001; ****, *P* < 0.0001. LOD indicates the limit of detection .

**Figure S12. AFAs enhance the competitiveness of CoNS against *S. aureus* in rich-medium biofilms.**

**A-H.** Wild-type *S. aureus* USA300 JE2 (WT) or its isogenic mutant defective for Lip1 and Lip2 (Δ*lip1/2*) was cultured alone or co-cultured with *S. simulans* or *S. epidermidis* for 24 h to form biofilms. Bacteria were grown in basic medium (BM), BM with 200 µM palmitoleic acid (PA), or BM with 200 µM PA and 200 µM palmitoleyl alcohol (POH). Biofilms were destroyed, serially diluted, and selectively plated for colony-forming unit (CFU) counting. CFU counts (**A, D, E,** or **F**) were used to calculate the relative abundance of *S. aureus* (WT or Δ*lip1/2*) and *S. simulans* (**B** or **G**) or *S. epidermidis* (**C** or **H**). Data shown are means ± SD for three biological replicates. Statistical significance was determined by two-way ANOVA with Tukey’s multiple comparisons test. *, *P* < 0.05; **, *P* < 0.01; ***, *P* < 0.001; ****, *P* < 0.0001. LOD indicates the limit of detection.

**Figure S13. Lipid-free medium abolishes the AFA tolerance of *S. simulans* in biofilms and drives competitive parity with *S. aureus*.**

**A-H.** Wild-type *S. aureus* USA300 JE2 (WT) or its isogenic mutant defective for Lip1 and Lip2 (Δ*lip1/2*) was cultured alone or co-cultured with *S. simulans* or *S. epidermidis* for 24 h to form biofilms. Bacteria were grown in chemically defined medium (CDM), CDM with 50 µM PA, or CDM with 50 µM PA and 50 µM POH. Biofilms were mechanically disrupted, serially diluted and selectively plated for colony-forming unit (CFU) counting. CFU counts (**A, B, D,** or **F**) were used to calculate the relative abundance of *S. aureus* (WT or Δ*lip1/2*) and *S. simulans* (**C** or **G**) or *S. epidermidis* (**E** or **H**). Data shown are means ± SD of three biological replicates. Statistical significance was determined by two-way ANOVA with Tukey’s multiple comparisons test. *, *P* < 0.05 ; **, *P* < 0.01; ***, *P* < 0.001; ****, *P* < 0.0001. LOD indicates the limit of detection.

**Figure S14. Increased susceptibility of *S. simulans* to AFAs in high-salt conditions.**

**A-B.** *S. epidermidis* (**A**) and *S. simulans* (**B**) were cultivated in basic medium (BM) or BM supplemented with 1.5% NaCl, or medium supplemented with increasing concentrations of palmitoleic acid (PA, from 25 to 200 µM) for 24 h. Area under the growth curves are shown in arbitrary units (AU).

**C.** *S. simulans* was cultured alone or co-cultured with WT *S. aureus* or Δ*lip1/2* mutant for 24 h to form biofilms. Bacteria were grown plain medium [BM or BM supplemented with 1.5% NaCl], medium with 100 µM PA, or medium with 100 µM PA and 100 µM POH. Biofilms were mechanically disrupted, serially diluted and selectively plated for colony-forming unit (CFU) enumeration. LOD indicates the limit of detection [log10(203 CFU/mL) = 2.3]. Data shown are means ± SD of three or four biological replicates. Statistical significance was determined by two-way ANOVA with Šídák’s multiple comparisons test (**A, B**) or with Tukey’s multiple comparisons test (**C**). *, *P* < 0.05; **, *P* < 0.01; ***, *P* < 0.001; ****, *P* < 0.0001.

**Figure S15. *S. epidermidis* maintains AFA resistance during high-salt stress.**

*S. epidermids* (**A**) was cultured alone or co-cultured with WT *S. aureus* (**B, C**) or Δ*lip1/2* mutant (**D, E**) for 24 h to form biofilms. Bacteria were grown in plain medium [basic medium (BM) or BM supplemented with 1.5% NaCl], medium with 100 µM PA, or medium with 100 µM PA and 100 µM POH. Biofilms were mechanically disrupted, serially diluted and selectively plated for colony-forming unit (CFU) enumeration. CFU counts (**A, B, D**) were used to calculate the relative abundance of *S. aureus* (WT or Δ*lip1/2*) and *S. epidermidis* (**C** or **E**). LOD indicates the limit of detection. Data shown are means ± SD of three or four biological replicates. Statistical significance was determined by two-way ANOVA with Tukey’s multiple comparisons test. *, *P* < 0.05; **, *P* < 0.01; ***, *P* < 0.001; ****, *P* < 0.0001.

**Figure S16. *S. aureus* invades preformed *S. simulans* biofilms in a lipid-dependent manner.**

Wild-type (WT) *S. simulans* or its isogenic Δ*ssL* mutant was cultivated alone in basic medium (BM) supplemented with 1.5% NaCl to form biofilms. After 24 h, non-adherent cells were removed before adding fresh medium (BM with 1.5% NaCl), or medium supplemented with 100 µM palmitoleic acid (PA) or 100 µM PA and 100 µM palmitoleyl alcohol (POH). For cocultivation experiments, WT *S. aureus* or Δ*lip1/2* mutant was added to preformed *S. simulans* biofilms. Following a further 24 h incubation with or without lipid supplementation, biofilms were mechanically disrupted, serially diluted and selectively plated for colony-forming unit (CFU) enumeration. CFU counts (**A, B, C,** and **D**) were used to calculate the relative abundance of *S. aureus* (WT or Δ*lip1/2*) and *S. simulans* (**E, F, G,** and **H**). LOD indicates the limit of detection. Data shown are means ± SD of three biological replicates. Statistical significance was determined by two-way ANOVA with Tukey’s multiple comparisons test. *, *P* < 0.05; **, *P* < 0.01; ***, *P* < 0.001; ****, *P* < 0.0001.
